# Evolution of higher mesenchymal CD44 expression in the human lineage: A gene linked to cancer malignancy

**DOI:** 10.1093/emph/eoac036

**Published:** 2022-08-30

**Authors:** Xinghong Ma, Anasuya Dighe, Jamie Maziarz, Edwin Neumann, Eric Erkenbrack, Yuan-Yuan Hei, Yansheng Liu, Yasir Suhail, Kshitiz, Irene Pak, Andre Levchenko, Günter P Wagner

**Affiliations:** Systems Biology Institute, Yale University, West Haven, CT 06516, USA; Department of Ecology and Evolutionary Biology, Yale University, New Haven, CT 06520, USA; Key Laboratory of Animal Cellular and Genetics Engineering of Heilongjiang Province, College of Life Sciences, Northeast Agricultural University, Harbin, China; Systems Biology Institute, Yale University, West Haven, CT 06516, USA; Department of Ecology and Evolutionary Biology, Yale University, New Haven, CT 06520, USA; Systems Biology Institute, Yale University, West Haven, CT 06516, USA; Department of Ecology and Evolutionary Biology, Yale University, New Haven, CT 06520, USA; Systems Biology Institute, Yale University, West Haven, CT 06516, USA; Systems Biology Institute, Yale University, West Haven, CT 06516, USA; Department of Ecology and Evolutionary Biology, Yale University, New Haven, CT 06520, USA; Cancer Biology Institute, Yale University, West Haven, CT 06516, USA; Department of Pharmacology, Yale Medical School, New Haven, CT 06510, USA; Cancer Biology Institute, Yale University, West Haven, CT 06516, USA; Department of Pharmacology, Yale Medical School, New Haven, CT 06510, USA; Department of Biomedical Engineering, University of Connecticut Health Center, Farmington, CT 06030, USA; Department of Biomedical Engineering, University of Connecticut Health Center, Farmington, CT 06030, USA; Systems Biology Institute, Yale University, West Haven, CT 06516, USA; Department of Ecology and Evolutionary Biology, Yale University, New Haven, CT 06520, USA; Systems Biology Institute, Yale University, West Haven, CT 06516, USA; Department of Biomedical Engineering, Yale University, New Haven, CT 06520, USA; Systems Biology Institute, Yale University, West Haven, CT 06516, USA; Department of Ecology and Evolutionary Biology, Yale University, New Haven, CT 06520, USA; Department of Obstetrics, Gynecology and Reproductive Sciences, Yale Medical School, New Haven, CT 06510, USA; Department of Obstetrics and Gynecology, Wayne State University, Detroit, MI 48202, USA

**Keywords:** CD44, cancer, gene regulation, malignancy, evolution, endometrium

## Abstract

CD44 is an extracellular matrix receptor implicated in cancer progression. CD44 increases the invasibility of skin (SF) and endometrial stromal fibroblasts (ESF) by cancer and trophoblast cells. We reasoned that the evolution of CD44 expression can affect both, the fetal–maternal interaction through CD44 in ESF as well as vulnerability to malignant cancer through expression in SF. We studied the evolution of *CD44* expression in mammalian SF and ESF and demonstrate that in the human lineage evolved higher *CD44* expression. Isoform expression in cattle and human is very similar suggesting that differences in invasibility are not due to the nature of expressed isoforms. We then asked whether the concerted gene expression increase in both cell types is due to shared regulatory mechanisms or due to cell type-specific factors. Reporter gene experiments with cells and *cis*-regulatory elements from human and cattle show that the difference of *CD44* expression is due to *cis* effects as well as cell type-specific *trans* effects. These results suggest that the concerted expression increase is likely due to selection acting on both cell types because the evolutionary change in cell type-specific factors requires selection on cell type-specific functions. This scenario implies that the malignancy enhancing effects of elevated CD44 expression in humans likely evolved as a side-effect of positive selection on a yet unidentified other function of CD44. A possible candidate is the anti-fibrotic effect of CD44 but there are no reliable data showing that humans and primates are less fibrotic than other mammals.

## INTRODUCTION

Cancer metastasis is the major cause of cancer-related mortality compared to the direct effects of the primary tumor. Malignancy rates differ greatly between mammalian species, broadly correlated with placenta type [1–3], where animals with lower placental invasion tend to be also less vulnerable to cancer malignancy [4]. Cancer progression and malignancy is in part a result of the interaction between tumor cells and the cancer-associated stroma consisting of cancer-associated fibroblasts (CAF), immune cells and extracellular matrix (ECM) [5]. In a previous study we have shown that bovine skin and endometrial stromal fibroblasts (SF and ESF, respectively) are less invasible by trophoblast and melanoma cells than their human counterparts and that a knockdown of *CD44* in human cells decreases their invasibility, i.e. increases their resistance to trophoblast and cancer invasion [6]. Here, we document the evolution of *CD44* expression in mammals from the boreoeutherian clade of placental mammals (roughly, primates, rodents, carnivores and hoofed animals) and investigate the genetic basis of expression differences between human and bovines, i.e. the contributions of *cis*- and *trans*-effects to species differences in *CD44* expression.

CD44 is a well-known membrane-bound receptor for ECM components that plays a major role in cancer progression and metastasis [8, 9]. CD44 expression in stromal fibroblasts is also important for the transformation of tissue fibroblasts into CAF, which aid tumor survival and local dissemination. The transformation of fibroblasts to CAF is caused by paracrine signals from the primary tumor through, including but not limited to, TGFb1, PDGF and osteopontin (OPN, aka SPP1) [10, 11]. Sharon and coworkers have shown that breast cancer cells secrete copious amounts of OPN and that tissue fibroblasts get transformed to CAF via the interaction of their CD44 and integrin α_v_β_3_ receptors [11]. These two receptors play partially overlapping roles in CAF transformation, where integrin causes primarily the migratory phenotype of CAF and CD44 their pro-inflammatory effects. The critical role for malignancy of stromal CD44 is further supported by a study that shows that mesenchymal stem cells require CD44 to be transformed into CAF in a conditioned media assay as well as *in vivo* recruitment to a tumor site [12]. In addition, the different isoforms of CD44 have different potential to affect malignancy rate, with the CD44s (CD44 standard) isoform being most strongly associated with malignancy [13]. Species differences in CD44 isoform expression in stromal cells could thus be a factor influencing the metastatic vulnerability of a species, and for that reason, we also compared the isoform expression in humans and cattle. In our previous study, we noted that human SF and ESF express higher levels of CD44 mRNA than cattle and the knockdown of CD44 transcripts in human cells make them less invasible by cancer and trophoblast cells [6].

Here, we show that high *CD44* expression in SF and ESF has specifically evolved in the primate lineage expressing the same dominant CD44s isoform in both cell types. The expression of *CD44* is regulated by homologous proximal *cis*-regulatory elements (CRE) in both cell types and species. Species differences in *CD44* expression are caused by both *cis*- as well as cell type-specific *trans*-factors. We also identify CEBPB as a SF-specific *trans*-factor explaining part of the higher *CD44* expression in human SF.

## RESULTS AND DISCUSSION

### Humans evolved high expression of CD44 in stromal cells

**Figure 1.**
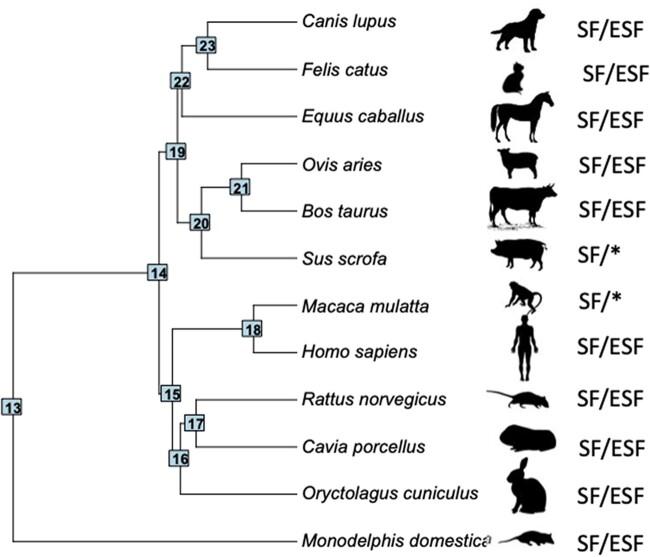
Taxon sample with phylogenetic relationships among 12 mammalian species based on the phylogeny of dos Reis *et al*. [7]. ‘SF/ESF’ labels next to species silhouettes indicate that both cell types, skin fibroblasts (SF) and endometrial stromal fibroblasts (ESF) could be cultured, and ‘SF/*’ indicates that only skin fibroblasts could be harvested from the respective species

To investigate gene expression evolution in SF and ESF, we cultured these two cell types from a sample mammalian species, including a marsupial, the gray short-tailed opossum (*Monodelphis domestica*), representing an outgroup to placental mammals, and nine species from the Boreoeutheria clade (roughly, primate and rodent as well as ungulates and carnivores) of placental (eutherian) mammals (see ‘Materials and methods’; Table 1; Fig. 1). For some species, we only were able to obtain SFs, macaque and domestic pig, and we include them in the analysis of gene expression in SFs.

**Table 1.** Samples for the comparative transcriptomic data

Species	Generic name	ESF biol	ESF exp	SF biol	SF exp	ESF raw data	SF raw data
*Homo sapiens*	Human	2	2	1	3	GSE87692	SUB6264591
*Canis lupus*	Dog	1	1	1	3	SUB8398078	SUB8398078
*Rattus norvegicus*	Rat	2	1	1	3	SUB8398078	SUB6229748
*Oryctolagus cuniculus*	Rabbit	3	1	3	1	SUB8398078	SUB6229748
*Monodelphis domestica*	Opossum	1	3	1	3	SUB8398078	SUB8398078
*Felis catus*	Cat	3	1	1	3	SUB8398078	SUB6229748
*Cavia porcellus*	Guinea pig	1	3	1	3	SUB8398078	SUB6229748
*Ovis aries*	Sheep	1	3	1	3	SUB8398078	SUB6229748
*Equus caballus*	Horse	3	1	3	1	SUB8398078	SUB6229748
*Bos taurus*	Cattle	1	2	1	3	GSE136299	SUB6229748
*Sus scrofa*	Pig	0	0	1	3	–	SUB8398078
*Macaca mulatta*	Monkey	0	0	3	1	–	SUB8398078

A biological replicate means that the cells are from different individuals, experimental replicate means that the whole experiment from growing the cells to sequencing has been repeated. Note that the term ‘technical replicate’ can mean various things from experimental replication to repeated sequencing of the same RNA sample, etc.

Gene expression was assessed by bulk RNAseq of isolated cultured fibroblasts and quantified as transcripts per million transcripts (TPM) [14, 15] based on one-to-one orthologous genes. Each gene was quantified using species-specific transcript reconstructions from the read data (see ‘Materials and methods’). Here, we focus on the results for *CD44*. A more comprehensive analysis of the transcriptome-wide data will be presented elsewhere.

**Figure 2.**
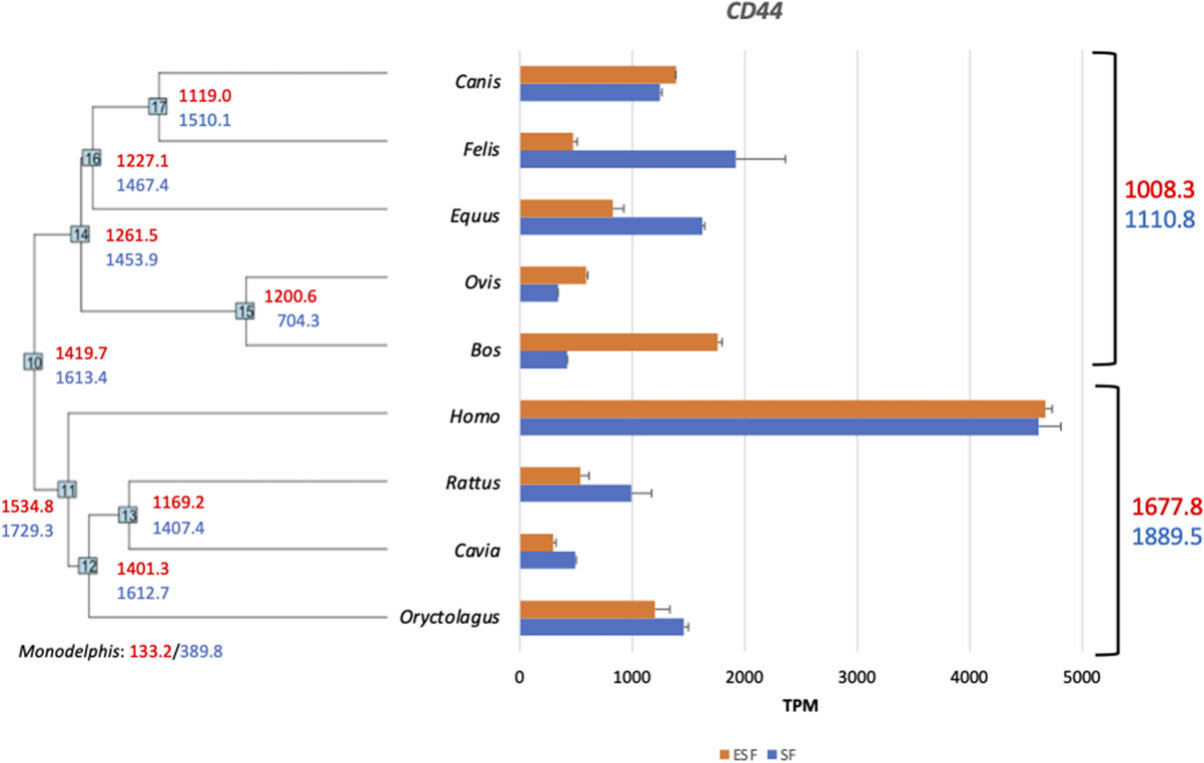
*CD44* expression in Boreoeutherian mammals. Gene expression levels (TPM) in skin (SF) and endometrial stromal fibroblasts (ESF) from nine Boreoeutherian mammals and the opossum *Monodelphis domestica* as an outgroup. At the nodes of the tree are the ancestral state reconstructions-based REML algorithm (see Materials and methods). Red, upper, numbers are the TPM in ESF and the blue, lower, numbers are for SF. On the right side are average expression number over the two sub-clades, the Laurasiatheria and the Euarchontoglires. Detailed explanation of replicates is given in Table 1: rabbit 2,2/1,3 (biol, exp ESF/biol, expSF); guinea pig 1,3/1,3; rat 2,1/1,3; human 2,2/1,3; cow 1,2/1,3; sheep 1,3/1,3; horse 3,1/3,1; cat 3,1/1,3; dog 1,1/1,3. The error bars are standard errors of the mean (A color version of this figure appears in the online version of this article.)

**Figure 3.**
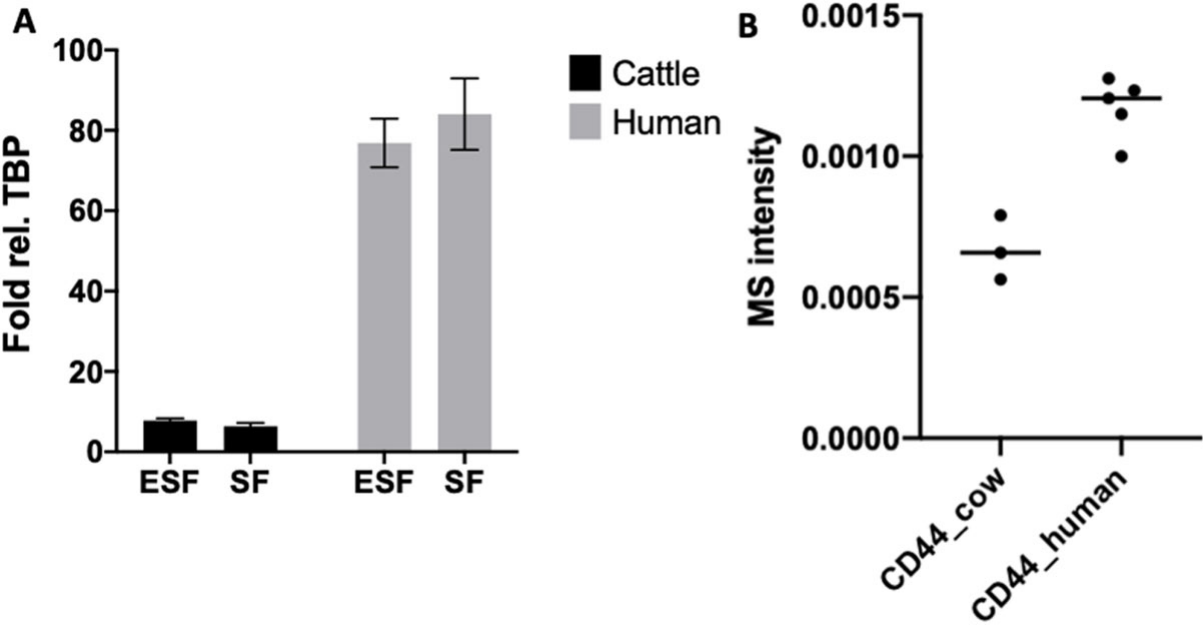
Comparison of CD44 expression between human and cattle. (**A**) qPCR confirmation of the high expression of *CD44* in human in comparison to cow [mean and SEM]. Experimental replicates: ESF = 13, SF = 9. (**B**) CD44 protein expression in skin fibroblasts from human and cow. Human skin fibroblasts express at least twice as much CD44 protein than cow skin fibroblasts

Figure 2 shows the expression levels of *CD44* mRNA in both SF and ESF for the 10 species included where we had both SF and ESF. At the nodes of this tree estimates of ancestral gene expression are noted based on the Residual Maximum Likelihood (REML) method [16, 17]. As can be seen from these data, the expression in all animals sampled, excluding humans, is below 2000 TPM (average w/o human 1062 TPM for SF and 885 TPM for ESF) and does not show very distinct evolutionary trends. However, in both human ESF and SF, the expression level is between 4600 and 4700 TPM. We confirmed this large discrepancy in *CD44* expression between human and bovine cells with qPCR (Fig. 3A, experimental replicates ESF = 13, SF = 9).

Interestingly, opossum has about a 10-fold lower expression level of *CD44* mRNA than even the non-human placental mammals, 133 TPM and 390 TPM in ESF and SF, respectively, suggesting an increase in expression associated with the evolution of placental mammals, coinciding with the evolution of invasive placentation [18–20].

**Figure 4.**
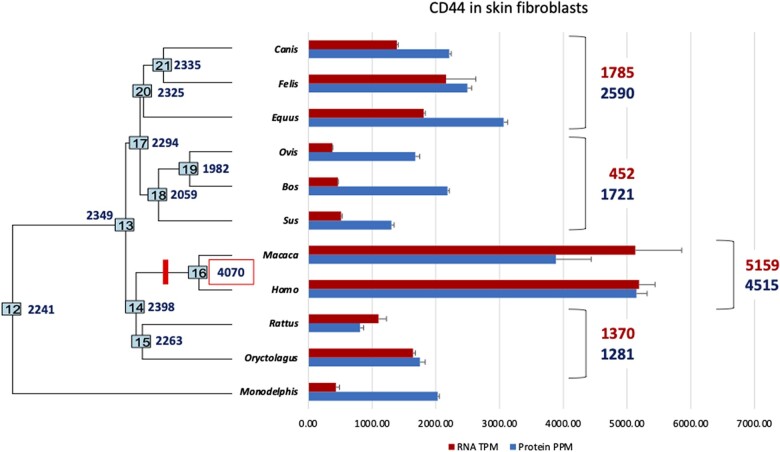
Evolution of CD44 RNA (red, above) and protein (blue, below) expression in skin fibroblasts as reconstructed from our 11 species sample. To the right, the averages for some of the clades are calculated for easier comparison. The numbers at the internal nodes represent ancestral state reconstructions of CD44 protein abundances in PPM. The bright red tick mark identifies a major evolutionary increase in CD44 protein expression. Note that the average RNA expression numbers slightly differ from those in Fig. 1 because of normalization to a different set of genes and species. RNA replicate numbers are reported in Fig. 2 and Table 1. The additional species are pig with one biological and three experimental replicates and monkey with three biological replicates. The proteomic data are based on three experimental replicates except for human where the number is five. Error bars represent standard errors of the mean (A color version of this figure appears in the online version of this article.)

Phylogenetic reconstruction of ancestral gene expression levels suggests that the common ancestor of Euarchontoglires had an expression level of ∼ 1535 TPM in ESF and 1729 TPM in SF, which implies a 3-fold increase of *CD44* expression in the primate lineage. For SF, for which we have also data from *Macaca mulatta*, we find that the level of *CD44* gene expression in monkey SF is indistinguishable from that in human SF (human = 5187 TPM, monkey = 5130 TPM, difference 1.1%; see Fig. 4). This suggests that the high expression of *CD44* in human SFs evolved before the most recent common ancestor of Catarrhini (OWM and apes) and after the ancestor of Euarchontoglires (primates, rodents and rabbits).

### Evolution of CD44 protein abundance

In order to investigate whether the RNA abundance differences between human and bovine SF are indicative of corresponding protein abundance differences, we first investigated the relative expression level of CD44 protein in human and cow SFs. Since protein quantification across species using antibodies is problematic due to species differences in the amino acid sequences of the target protein, we resorted to a new and reproducible quantitative mass spectrometry (MS) technique (see ‘Materials and methods’). We compared the ratio of CD44 MS quantities from each sample to the total protein signals in the MS run. The reference set of proteins for each sample was the overlap between the proteins detected in human and bovine samples (6652 and 5510 proteins for human and cow, respectively, with an overlap of 4269 proteins) to assure commensurable expression scales for human and cow samples (Fig. 3B). The relative CD44 protein abundance in bovine SFs was lower than that in human samples, as expected (t-test *P* = 7.25 10^−3^). The fold difference of human compared to cattle was 2.05×, which is smaller than the fold differences based on RNAseq and qPCR (10.9× and 13.1×, respectively). Whether this differences in RNA versus protein abundances are due to regulation at the post-transcriptional level or due to post-translational modifications affecting peptide identification by MS is not clear.

It is well known that the evolution of RNA expression levels and protein levels is not strictly correlated [21–24]. In order to assess the degree to which the evolution of *CD44* RNA expression relates to CD44 protein expression, we performed mass spectroscopy quantification on SFs from a taxon sample of 11 species. This analysis includes additional SF data from pig (*Sus scrofa*) and macaque (*Macaca mulatta*) but lost our guinea pig sample (Fig. 4).

Over the 11 species, the Spearman correlation between RNA and protein expression in SF is 0.7545 (Supplementary Fig. 1), relatively high compared to the average over all peptides detected (<0.3, Ba *et al*., in press). Nevertheless, both humans and monkeys have the highest RNA and protein expression levels in our taxon sample (Fig. 4), much higher than rat and rabbit, their closest relatives in this sample. Within the Laurasiatheria, cloven hoofed animals (Artiodactyla, pig, sheep and cattle) have the lowest RNA and protein expression, although the RNA is much lower compared to carnivores and horse than the protein abundance. Notable is also the low RNA expression in opossum, while its protein expression is comparable to eutherian species, except primates. Phylogenetic analysis reveals one significant event in the evolution of CD44 protein abundance in SFs in primates, specifically after the node 14 and before node 16, i.e. the most recent common ancestor of old world monkeys and apes (Catarrhini, bright red tick mark in Fig. 4).

### *CD44* splice isoform expression

*CD44* is known to express a large number of splice isoforms which play different biological roles and have different effects on malignancy rate [8, 9, 13]. Here, we investigate the expression of isoforms from our sequence read data in human and cattle ESF and SF in order to assess whether the differences in invasibility of cattle and human fibroblasts could also be influenced by the kind of isoforms expressed by cattle and human cells.

**Figure 5.**
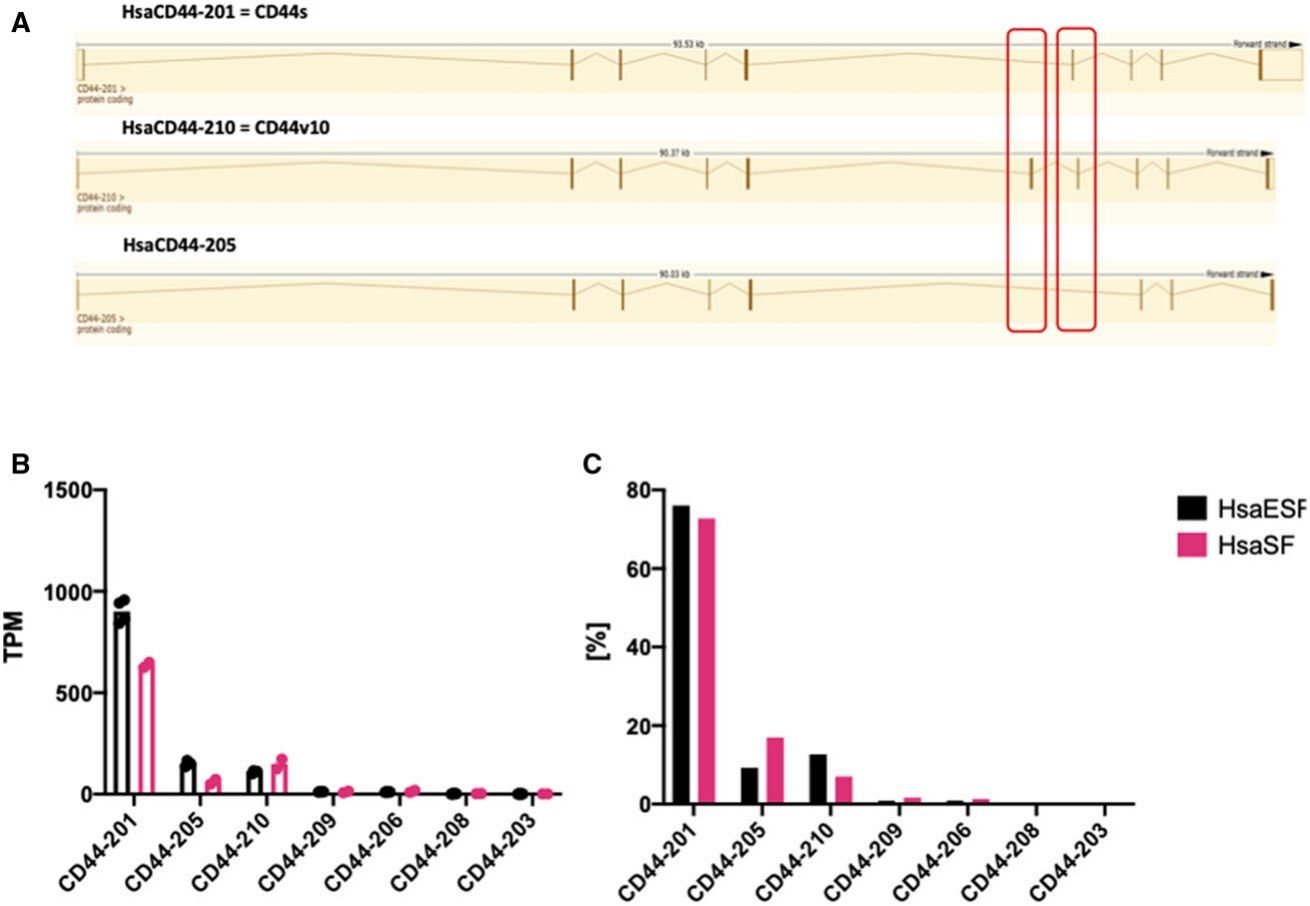
*CD44* RNA isoform expression in human skin and endometrial fibroblasts. (**A**) Intron–exon structure of the three dominant isoforms from human mesenchymal cells: HsaCD44-201 which is also known as CD44s, HsaCD44-210 which retains the variable exon 10, Hsav10, and HsaCD44-205 which lacks one of the ‘constant’ exons. The red boxes indicate the position of variable exons [images courtesy of ENSEMBL.org]. (**B**) Expression levels [TPM] of isoforms in SF and ESF. Note that only three isoforms have considerable levels of expression [mean and SEM]. (**C**) Relative expression levels [%] of CD44 isoforms in human skin and endometrial fibroblasts (A color version of this figure appears in the online version of this article.)

In humans, the *CD44* gene has been described as having 19 exons divided into 9 constant exons 1–8 and 10 (‘exon’ 9 is reported to be never expressed) and nine so-called variable exons, called v2–v10 [25]. In quantifying isoform abundances, we focused on high-quality protein-coding transcript annotations that correspond to ENSEMBL consensus coding sequences (CCDS) and we refer to them by using the ENSEMBL transcript names with the format HsaCD44-2xx. More than 96% of transcripts belong to three isoforms: HsaCD44-201, HsaCD44-210 and HsaCD44-205 (Fig. 5A), where the by far most dominant isoform is HsaCD44-201 with 9 exons and 361 amino acids (Fig. 5B and C). In the literature, this isoform is sometimes called the standard isoform, CD44s. HsaCD44-210 is identical to HsaCD44-201 with the addition of the most 3′ of the variable exons, ENSE00003608645 = v10, and can thus be called CD44v10 following a naming tradition in the biomedical literature (Fig. 5A). Not much is known about the functional roles of this specific isoform. The third transcript, HsaCD44-205, is unusual as it has eight exons and is lacking one of the so-called ‘constant’ exons of HsaCD44-201/CD44s, namely ENSE00003526469 (Fig. 5A and Supplementary Fig. 2A). Differences in the non-coding sequences in annotated 3′ and 5′ exons are not affecting the predicted amino acid sequence (Supplementary Fig. 2B).

**Figure 6.**
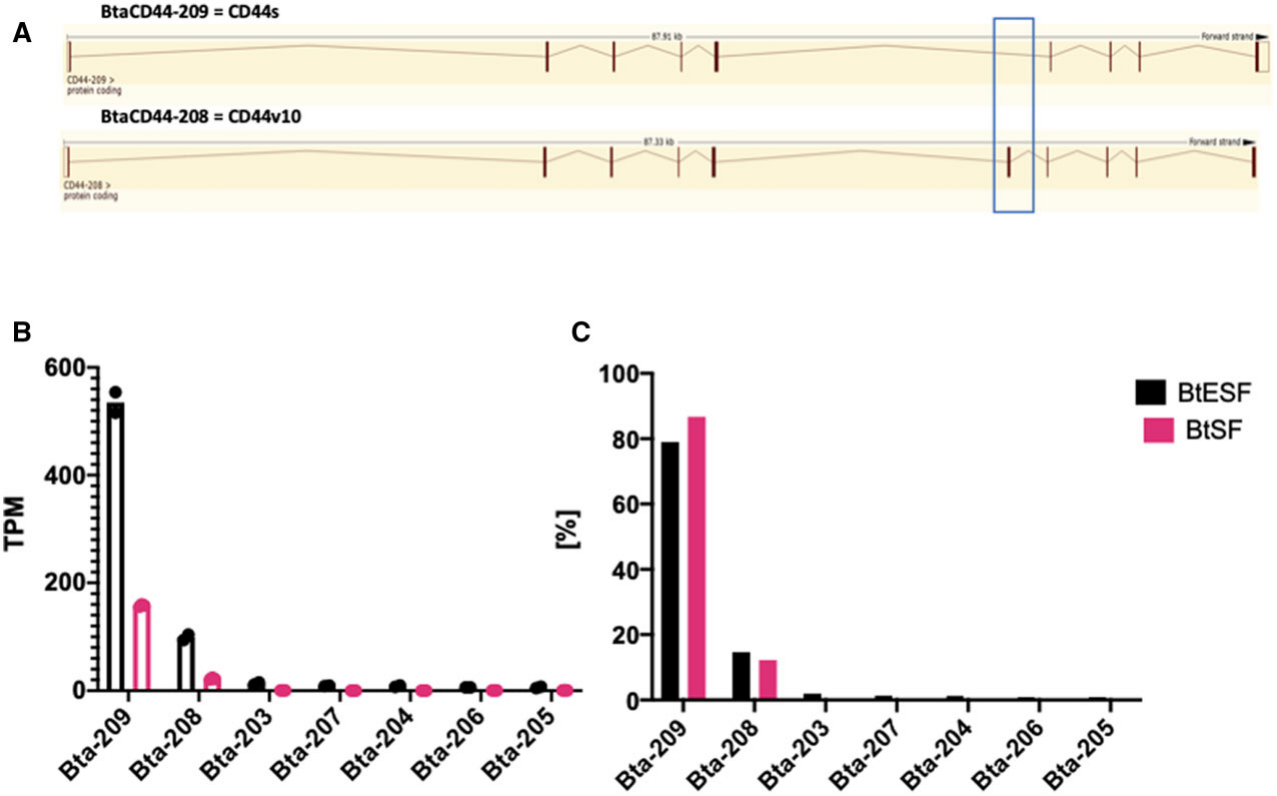
*CD44* RNA isoform expression in bovine skin and endometrial fibroblasts. (**A**) intron–exon structure of the two dominant isoforms from bovine mesenchymal cells: BtaCD44-209 which is also known as CD44s, BtaCD44-208 which retains the variable exon 10 like the human HsaCD44-210 (see Fig. 5A). The blue box indicates the position of the variable exon [images courtesy of ENSEMBL.org]. (**B**) Expression levels [TPM] of isoforms in SF and ESF. Note that only two isoforms have considerable levels of expression [mean, SEM]. (**C**) Relative expression levels [%] of CD44 isoforms in bovine skin and endometrial stromal fibroblasts (A color version of this figure appears in the online version of this article.)

In bovine cells, the majority of transcripts belong to two isoforms, BtaCD44-209 and BtaCD44-208, which together make up >93% of transcripts in ESF and 98.9% in SF (Fig. 6A and B and Supplementary Fig. 2C). In both cell types, the dominant transcript is BtaCD44-209 with 79% and 87% representation, respectively (Fig. 6C). Note that the ENSEMBL transcript numbers in different species are not indicative of homology. Inspection of the annotated sequences revealed that BtaCD44-209 is homologous to HsaCD44-201 and is thus a bovine homolog of human CD44s (Supplementary Fig. 3), and BtaCD44-208 is homologous to HsaCD44-210 (Supplementary Fig. 4), which we call CD44v10.

In both species and cell types, the most abundant protein coding transcript is CD44s (HsaCD44-201 and BaCD44-209), with a minor contribution of CD44v10 (HsaCD44-210 and BtaCD44-208). In addition, human cells express a smaller isoform of eight exons, HsaCD44-205, which is not much discussed in the literature. Hence, human stromal cells differ from cow stromal cells by both an overall higher expression of CD44 (Fig. 2) and the presence of a minor isoform, HsaCD44-205 (Figs 5 and 6), while the relative abundance of the two major isoforms is similar in SF and ESFs in both species. This suggests that the main effect of CD44 on invasibility of cattle and human fibroblast [6] is due to differences in expression level of CD44 rather than the nature of the dominant isoform.

### *CD44* is transcribed from the same promoter in skin- and endometrial fibroblasts

The RNAseq and qPCR results showed that the primate lineage experienced an increase in *CD44* RNA expression in both cell types, SF and ESF (Figs 2 and 3). A concerted change in gene expression can be explained either by the fixation of pleiotropic mutations that affect gene expression in both cell types [26, 27] or by natural selection acting on gene expression in both cell types likely leading to different mechanisms of expression control in the two cell types. On the mechanistic level, pleiotropic change in gene expression can either be due to mutations in CRE for *CD44* that are active in both cell types or changes in the activity of *trans*-factors that affect *CD44* expression in both cell types. We investigate these two possibilities (*cis*- and *trans*-regulation) in this and the next sections.

To investigate whether the evolution of *CD44* gene expression can be due to changes in the same *cis*-regulatory element or by species alternative specific promoters, we first asked whether the *CD44* RNA was transcribed from the same promoter in both cell types and species. We performed a 5′ RACE experiment on cDNA from both cell types and species and found that the 5′ RACE fragments recovered are of similar length (∼460 bp, with about a ∼120 bp 5′ UTR) (Supplementary Figs 5A and 6B) and amplify orthologous sequences (Supplementary Fig. 5B). We note that the 5′ UTRs reported in annotated transcript sequences in ENSEMBL differ greatly in length from the 5′ UTR that we amplified (see above). For instance, the 5′ UTR of HsaCD44-201 is reported to be 434 bp. We could not trace the tissue source from where the HsaCD44-201 RNA was isolated, but the results suggest that the cDNA was cloned from another cell type than those investigated here, suggesting that *CD44* in cell types other than skin and endometrial fibroblasts are controlled by an alternative promoter.

From these results, we concluded that the promoters used in the two mesenchymal cell types, SF and ESF, are the same and are likely different from that of other cell types. It is possible that the proximal CRE shared between SF and ESF can be responsible for the concerted increase in CD44 expression in the human fibroblasts. We tested this possibility with reporter gene experiments.

### Proximal promoter regions recapitulate species and cell-type differences in reporter gene experiments

In order to measure the contribution of proximal *cis*- and *trans*-factors to the gene expression difference between bovine and human, ∼3 kb fragments were cloned upstream from the TSS from the bovine and human *CD44* locus’ TSS into a reporter construct (see ‘Materials and methods’, experimental replicates SF = 5, ESF = 8). We call these fragments pCRE for ‘proximal Cis-Regulatory Element’ in order to emphasize that we did not test distal enhancers. We chose a fragment of 3 kb for testing because the majority of experimentally confirmed transcription factor binding sites influencing *CD44* expression are located within 2 kb of the transcriptional start site (Supplementary Table 1). Hence our experiments reported here test the influence of species difference in the sequence of the proximal promoter. More distal enhancers have been described [28] but are not tested here.

**Figure 7.**
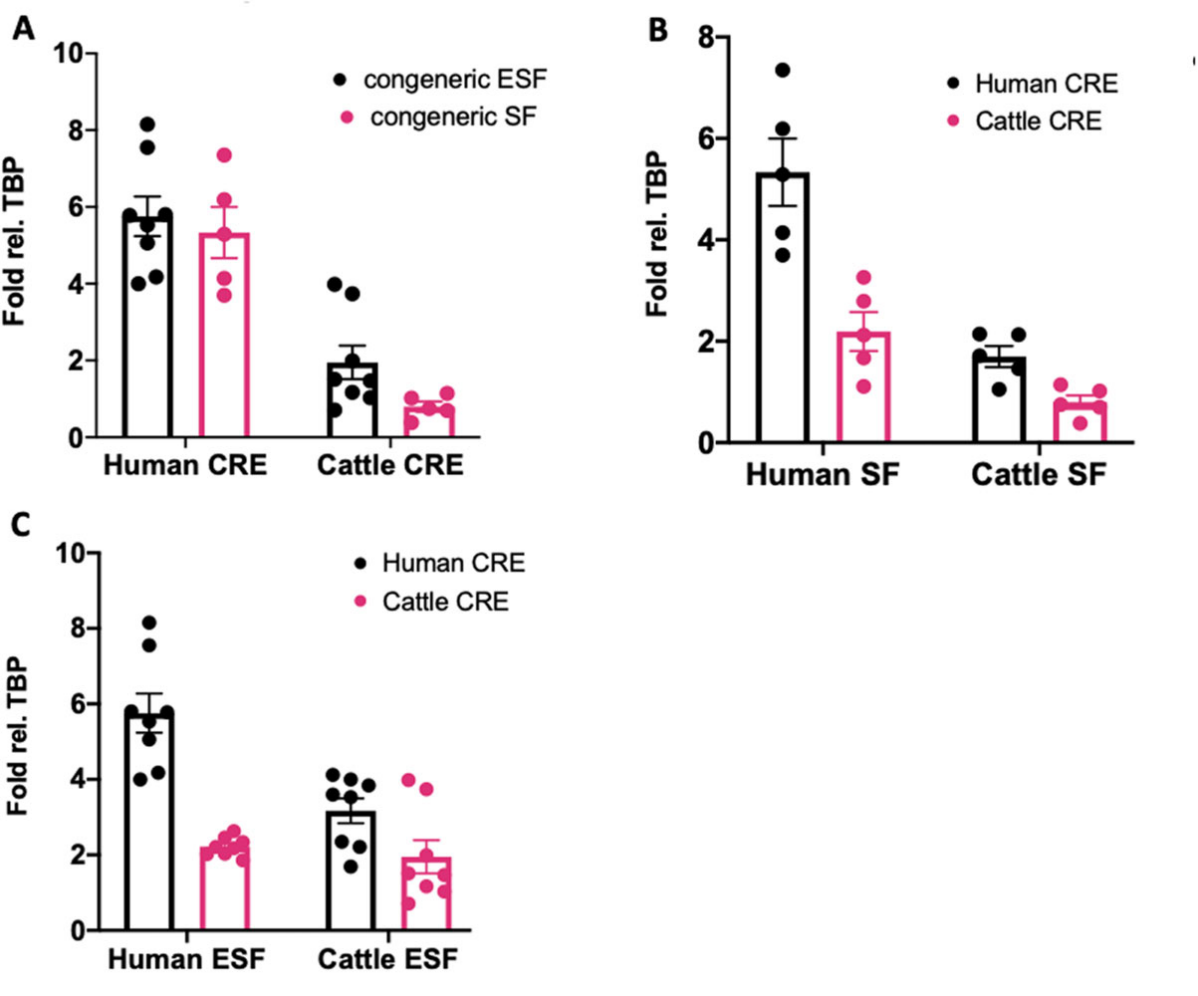
Results of reporter gene expression experiments with human and bovine *CD44* promoters in human and bovine skin and endometrial fibroblasts. (**A**) Expression of reporter gene transcripts [qPCR fold difference to TBP internal reference] of human and bovine promoters in their cognate cells. Note that the pattern of expression qualitatively reproduces the cell type and species typical expression levels found in RNAseq (see Fig. 2). (**B**) Cross species comparison of promoter activity in skin fibroblast cells. Black bars for human promoter, pink for bovine promoter. Note that the bovine promoter drives lower gene expression than the human promoter suggesting that some of the differences in human and bovine gene expression are caused by differences in promoter sequences. In addition, both promoters drive higher gene expression in human cells compared to bovine cells suggesting that *trans*-regulatory differences are contributing to differences between bovines and humans. (**C**) Cross species comparison of promoter activity in endometrial fibroblasts. The annotation is analogous to (B) and the results also suggest both *cis*- as well as *trans*-regulatory factors contribute to species differences in *CD44* expression (A color version of this figure appears in the online version of this article.)

We compared the reporter gene RNA expression driven by the human and bovine pCRE in their cognate cell types (Fig. 7A). The activity of the human pCRE in human ESF and SF was higher than the bovine pCRE in bovine cells (fold differences for ESF and SF: 3.0× and 6.7×, respectively; t-test of lnFOLD difference *P* = 4.5 10^−4^ and *P* = 7.5 10^−5^, respectively). Furthermore, the activity of the human pCRE in the two human cell types is statistically indistinguishable (*P* = 0.671), but in the bovine cells, the activity of the bovine pCRE is lower in SF than in ESF (2.4×; *P* = 1.8 10^−2^). We conclude that the activity of the reporter system qualitatively recapitulates the pattern seen in the intrinsic *CD44* RNA expression measured by RNAseq or qPCR among species and cell types, including the lower expression in bovine SF compared to bovine ESF (Figs 2 and 3).

### Both promoter differences and *trans*-regulatory factors contribute to species and cell-type differences

Next, we considered the activities of the human and bovine pCRE in all combinations of cell types and species of origin with the aim of assessing the contributions of both *cis*- and *trans*-regulatory effects (Fig. 7B and C). *Cis*-regulatory effects are those caused by differences in the promoter region, i.e. the number and strength of transcription factor binding sites, while *trans*-effects are those caused by the cellular environment, i.e. the expression of transcription factors, transcriptional co-factors and protein-modifying enzymes such as kinases or other protein-modifying enzymes affecting transcription factors. A two-way ANOVA on the log transformed fold values for SF reveals strong evidence for both *cis*- as well as *trans*-effects (*P* = 8.0 10^−5^, F = 27.73, 1/16 dgf for the *cis*-effect, and *P* = 6.25 10^−6^, F = 43.39, 1/16 dgf for the species *trans*-effect among SF) with similar effect sizes (on average 2.28-fold *cis*-effect and 2.94-fold *trans*-effect). Similar results were found for ESF. The significance levels of *cis*- and *trans*-effects in ESF were 2.73 10^−6^ (F = 34.24, 1/28 dgf) and 1.88 10^−3^ (F = 11.77, 1/28 dgf), respectively. An interesting detail is that the size of the *cis*-effect is comparable in both cell types (2.28× for SF and 2.05× for ESF), while the *trans*-effects are different by a factor 2 (2.94× in SF and 1.44× in ESF) hinting at cell type-specific *trans*-regulatory landscapes. An unexpected result was that in both cell types, there was no interaction effect between *cis*- and *trans*-regulatory factors in spite of strong direct effects.

We next performed a three-way ANOVA with three factors: species of cell origin, cell type, SF or ESF, and species of promoter origin with log Fold change as the response variable (Table 2). The results provide strong evidence for species, cell type and *cis*-regulatory effects, like the separate two-way ANOVAs for each cell type. The *P*-values for all the direct effects are between 10^−10^ and 10^−3^. In addition, there is a significant interaction effect between species and cell type (*P* = 4.7 10^−3^; F = 8.87, 1/44 dgf). The latter further supports the conclusion that gene regulation of *CD44* is strongly influenced by species-specific *trans* regulatory factors, suggesting that *CD44* expression evolution is happening in both cell types due to the evolution of cell type-specific evolutionary changes, rather than mutations that affect both cell types.

**Table 2.** Three-way ANOVA of the reporter gene experiments with human and cow pCRE in human and cow skin and endometrial fibroblasts

Source of variation	*SS*	*d.f.*	*MS*	*F*	*P*-level
*Factor #1 (species of cell)*	6.21209	1	6.212	45.60	2.64E−08
*Factor #2 (promoter)*	8.39617	1	8.396	61.63	6.62E−10
*Factor #3 (cell type)*	1.88854	1	1.889	13.86	5.60E−04
*Factor #1 + #2 (species of cell × promoter)*	0.2093	1	0.209	1.54	2.22E−01
*Factor #1 + #3 (species of cell × cell type)*	1.2089	1	1.209	8.87	4.69E−03
*Factor #2 + #3 (promoter × cell type)*	0.02672	1	0.027	0.20	6.60E−01
*Factor #1 + #2 + #3 (species of cell × promoter × cell type)*	0.02914	1	0.029	0.21	6.46E−01
*Within groups*	5.99452	44	0.136		
*Total*	23.96538	51	0.470		

The response variable is the log-transformed fold difference measured by qPCR.

### Evolution of *CD44* regulation

A review of the role of *CD44* in cancer biology has summarized known upstream regulatory factors for *CD44* expression in cancer cells [9]. Positive regulators are SP1, EGR1, TCF4, AP-1, NSFKB and ETS-1. Negative regulators are p53, KLF4 and FOXP3. In order to find a mechanistic explanation for the high *CD44* expression in human mesenchymal cells, we looked for positive regulators that are higher expressed in human cells and negative regulators that are higher expressed in bovine cells in our transcriptomic data (Supplementary Fig. 7). The positive transcription factors with higher expression in humans are *SP1, TCF4* and *NFKB* (Supplementary Fig. 7A). We tested all three of them with siRNA-mediated knockdown experiments in human ESF and did not detect any change in *CD44* expression (Supplementary Fig. 8A). Among the negative regulators, only one has a bovine-biased expression, *KLF4* (Supplementary Fig. 7B). We tested the effect of KLF4 KD in bovine SF and ESF and could not detect any upregulation of *CD44* in these cells (Supplementary Fig. 8B). We conclude that the regulators summarized in Chen *et al*. (2018), do not include the *trans*-regulatory factors responsible for *CD44* expression in human and bovine fibroblasts.

To find *cis*-regulatory changes that could explain the high *CD44* expression in human mesenchymal cells, we performed a preliminary transcription factor binding sites search in the 5 kb upstream of the CD44 locus from human, rabbit, guinea pig, rat, horse, sheep and cattle and identified transcription factors with binding site abundance in human higher than any of the other species and expression levels in both human ESF and SF of ≫3TPM (Supplementary Table 2). The reason we focused on the proximal region is that all but one of the experimentally confirmed transcription factors bind to a region closer than 1.42 kb upstream of the TSS of CD44 (Supplementary Table 1). This search revealed six candidate transcription factors: CEBPB, ZNF410, E2F7, ATF1, CEBPG, NR4A1 (Supplementary Fig. 9). Among them, we chose CEBPB for further investigation. CEBPB is a well-known transcription factor essential for the differentiation of human decidual and other mesenchymal cell types such as adipocytes. RNA expression of CEBPB is ∼ 95 TPM in both human cell types.

**Figure 8.**
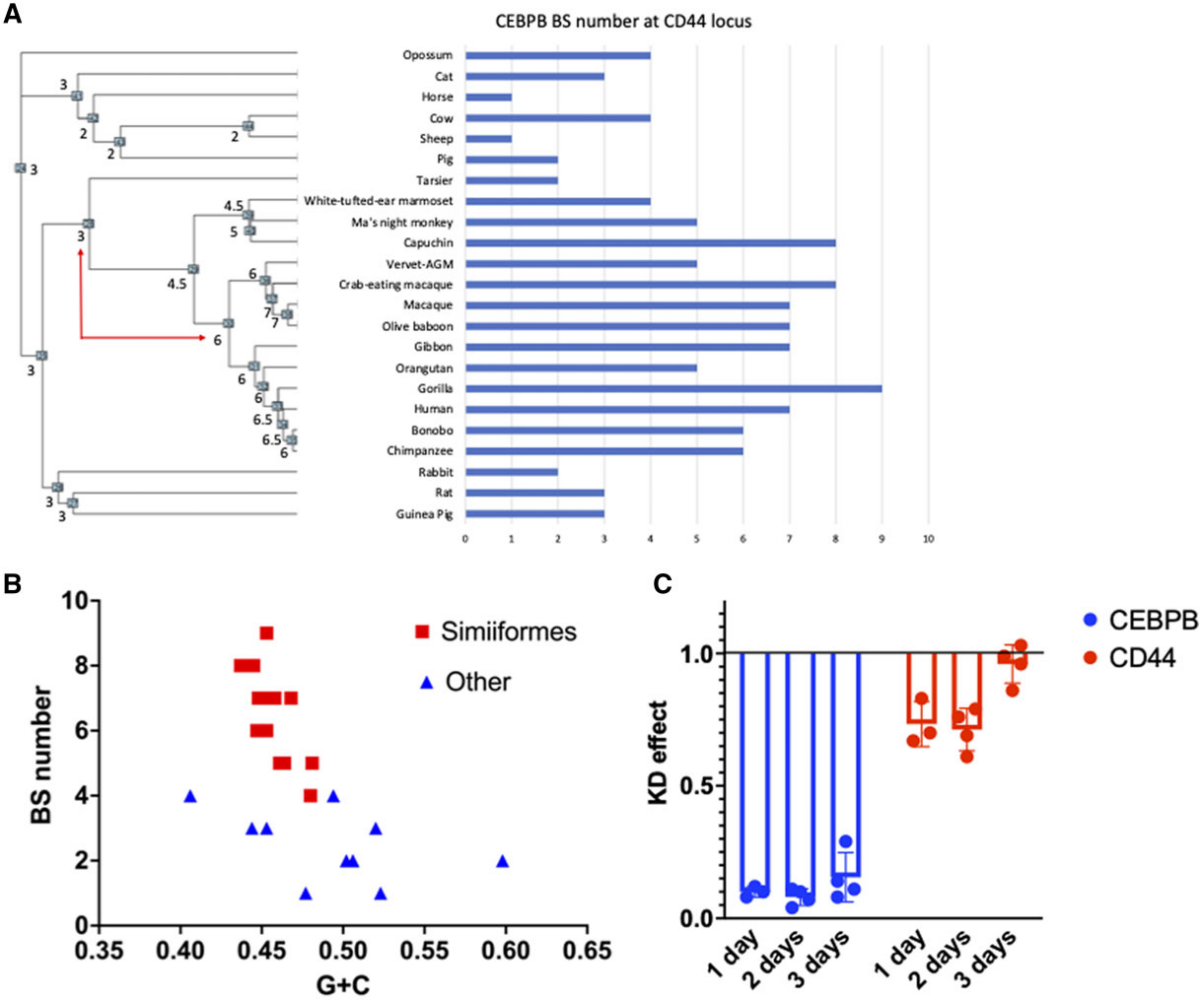
Evolution of CEBPB regulation of *CD44* in fibroblasts. (**A**) Evolution of CEBPB-binding site numbers at the *CD44* locus. (**B**) Relationship between base composition and binding site numbers. (**C**) Effect of CEBPB KD on *CD44* expression in human skin fibroblasts (experimental replicates = 4; one replicate at 1 day with insufficient KD of CEBPB was eliminated)

In order to investigate whether CEBPB regulation can be a factor in explaining the high *CD44* expression in humans, we performed a more detailed exploration of CEBPB-binding site evolution at the proximal genomic region of the *CD44* locus. In this search, we included genomic sequences from 23 mammalian species, with 13 primate species to assess if and when in primate evolution CEBPB-binding site numbers increased. Using the JASPAR position weight matrix MA0466.2 and a log-likelihood threshold of 7, we screened a 6000-bp region around the *CD44* TSS (–5000/+1000 bp). The estimated transcription factor binding site numbers vary between 1 in sheep and 9 in gorilla (Fig. 8A). A maximum parsimony reconstruction with an ordered character model revealed that all the nodes outside the Simiiformes (NWM, OWM and Hominoidea, aka apes) had an inferred binding site number of 2 or 3, with the lower number limited to hoofed animals. The ancestral node of the Catarrhini (OWM and Hominoidea) has an inferred binding site number of 6 or 7. The node uniting NWM and Catarrhini (Simiiformes) has an inferred binding site number between 4 and 5. The inferred binding site numbers increase 2-fold between the ancestral node of Haplorrhini (Tarsier and Catarrhini) to the ancestral node of Catarrhini, consistent with our finding that *CD44* expression in SFs increased before the most recent common ancestor of Catarrhini (see Fig. 4).

We then investigated whether the difference in binding site numbers can be explained by differences in base composition, rather than specific evolution of binding sites (Fig. 8B). We determined the G + C content of the promoter region and found a range of base composition between 0.4 and 0.6 G + C content. This range is covered in its entirety by the 10 species in our taxon sample that are not Simiiformes (i.e. not NWM, OWM or apes), and the binding site number in these species varies between 1 and 4. The range of G + C content for Simiiformes is 0.44–0.48 and their binding site numbers vary from 4 (white-eared Marmoset, a NWM) to 9 (gorilla). This pattern suggests that the increase in binding site number in higher primates (Simiiformes) is not caused by a secular change in base composition (Fig. 8B).

To assess the causal relevance of CEBPB regulation in human SFs, we were using a siRNA knockdown approach. The KD efficiency was high, at ∼90% reduction on average; one replicate with KD efficiency lower than 80% was ignored. In human ESF, the CEBPB KD did not affect *CD44* expression, but in SF, we found a consistent reduction of ∼30% of *CD44* expression at Days 1 and 2 after KD but no effect on Day 3 (Fig. 8C). Furthermore, the results suggest that the loss of CEBPB after KD is compensated by other factors over a time period of 3 days *in vitro*. We interpret these results as indicating that CEBPB is relevant for *CD44* expression in human SFs but not in human ESF, consistent with the finding above that ESF and SF diverged in their *trans*-regulatory landscape, as shown in the reporter gene assays (see above). These results suggest that in the primate lineage, CEBPB may have been added on to the ancestral gene regulatory network for *CD44* in fibroblast. Hence its role is likely modulatory rather than essential for *CD44* expression.

The influence of CEBPB on *CD44* expression accounts for ∼30% of the expression in human SFs. Given the distribution of transcription factor binding sites, we suggest that CEBPB is in part responsible for the lineage-specific increase in CD44 expression in the primate SFs. The lineage-specific increase of *CD44* expression is about 3× (Fig. 2), and thus the recruitment of CEBPB explains about half of the increase in the primate lineage. But we note that CEBPB only explains differences in gene expression in SFs but not in endometrial fibroblasts and thus supports the conclusion that evolution of *CD44* expression was realized, in part, by cell type-specific evolutionary changes in gene regulation.

## CONCLUSIONS

Higher *CD44* expression causes higher stromal invasibility of fibroblast populations by cancer cells [6], and may thus, in part, explain the high vulnerability of humans to malignancies of the skin compared to that of bovines and horses [1, 2]. We could exclude the possibility that the higher invasibility of human fibroblast populations is due to species-specific CD44 isoform expression as the dominant isoform expressed in bovine and human fibroblasts is the same. We conclude that the higher invasibility of primate fibroblasts attributable to CD44 is primarily due to higher expression level rather than the nature of the expressed isoform. In addition, the primate lineage evolved an increased expression of *CD44* in SFs in concert with endometrial fibroblasts, while the expression in non-primates remains ∼3–4-fold lower and does not display distinct evolutionary trends or correlated expression between cell types.

A change in the expression of *CD44* in the primate lineage can be caused either by directional selection or by relaxed stabilizing selection. We did not perform a formal statistical test for directional selection because we do not have estimates for the mutational variance, *V_m_*, for *CD44* expression which is necessary to test for selection on a quantitative character [29]. In addition, it is well understood that tests based on quantitative genetic theory have low power to detect directional selection, because random drift in most cases is strong enough to explain larger divergence than detected between species [30, 31]. Nevertheless, random genetic drift is not a plausible explanation for the observed pattern. At the one hand, the changes in *CD44* expression is the same in both cell types, and thus unlikely to be random. Also, the fact that in SF, the expression levels are the same in humans and monkeys are hard to explain by random drift.

An investigation of the differences in gene expression levels in cattle (low *CD44* expression) and human (high *CD44* expression) shows that cell type-specific changes in gene regulation make a substantial contribution in addition to changes in shared CRE. These results suggests that the correlated evolution of *CD44* expression in SF and ESF was driven by selection on *CD44* function in both cell types, rather than due to correlated effects from selection on only one cell type and pleiotropic effects on gene expression in the other cell type. For correlated selection response, one would predict that the evolutionary change is different between the two cell types. Quantitative genetic theory predicts that the trait under direct selection changes to a larger degree than the character that only shows a correlated selection response [32], because the genetic correlation between the traits in general is < 1. But since the *CD44* expression is the same in SF and ESF, correlated selection response is not a tenable explanation. In sum, the only plausible explanation is that natural selection acted simultaneously on both cell types to increase *CD44* expression.

The scenario of simultaneous selection for higher *CD44* expression in both cell types raises the question what the functional relevance of *CD44* expression could be for these cell types. Of note is that the dominant *CD44* isoform is the same in both cell types and thus CD44 likely can play the same biological role. However, what this function could be remains unclear. One possibility is that the primate lineage experienced selection in favor of anti-fibrotic phenotype in both stromal cell types, as CD44 can interact with the TGFb1 receptor inducing an anti-fibrotic phenotype [33]. Increased CD44 expression could affect wound healing in the skin as well as non-scarring regeneration of the endometrium after menstruation or birth. Incidentally, both, menstruation as well as increased *CD44* expression, evolved before the most recent common ancestor of apes and old-world monkeys [34]. It is intriguing to note that the only rodent species known to menstruate, the spiny mouse (*Acomys cahirinus*) [35], is also known for extreme levels of skin regeneration ability [36]. The increased vulnerability to cancer metastasis in humans could thus be a negative pleiotropic effect driven by selection for an anti-fibrotic phenotype. The cancer-related phenotype of increased malignancy would predominantly affect later life stages and would therefore have limited impact on fitness, as predicted by life history theory [37]. Hence the evolution of *CD44* expression and its associated cost in terms of cancer malignancy would be likely a case of antagonistic pleiotropy, where positive selection in early life stage is associated with disease burden in older age.

## MATERIALS AND METHODS

### Cell sourcing

For the non-human cells, we established primary cell lines for both ESF and SF (for details, see below). We aimed at obtaining three biological replicates (defined as representing different individuals), but was not feasible in all cases because of contamination or poor cell survival or accessibility of specimen (see overview in tables).

#### Human endometrial stromal cells and skin fibroblasts

An immortalized human ESF cell line was obtained from the Gil Mor group and two primary cell lines from clinical biopsies from Day 3 and 9 of the menstrual cycle, respectively. Both were obtained during polypectomy and neither woman took oral contraceptives. Human SFs (BJ5ta) were purchased from ATCC (CRL-4001).

#### Skin fibroblasts

Cow (*Bos taurus*), dog (*Canis lupus*), guinea pig (*Cavia porcellus*), horse (*Equus caballus*), cat (*Felis catus*), monkey (*Macaca mulatta*), opossum (*Monodelphis domestica*), rabbit (*Oryctolagus cuniculus*), sheep (*Ovis aries*), rat (*Rattus norvegicus*), and pig (*Sus scrofa*) SFs were obtained from fresh skin tissue. A small piece of skin was collected, hair removed and the sample was washed in PBS buffer and cut into strips ∼ 1.0 cm^2^. Dermis was separated from epidermis by enzymatic digestion (35 min in 0.25% Trypsin buffer at 37°C, followed by dissociation buffer (1 mg ml^–1^ collagenase, 1 mg ml^–1^ Dispase, 400 μg ml^–1^ DNase I) for 45 min at 37°C). Epidermis was removed, 2 mm pieces were cut from the dermis and transferred to a 12-well plate and covered with media. Fibroblasts emerged from the explants and grew to confluency in growth media. Extra tissue was removed.

#### Endometrial stromal fibroblasts

Cow (*Bos taurus*), dog (*Canis lupus*), guinea pig (*Cavia porcellus*), horse (*Equus caballus*), cat (*Felis catus*), monkey (*Macaca mulatta*), opossum *(Monodelphis domestica*), rabbit (*Oryctolagus cuniculus*), sheep (*Ovis aries*), rat (*Rattus norvegicus)*, *and* pig (*Sus scrofa*) ESFs were obtained as follows. Uterine tissues were collected from each species and primary ESFs were obtained by enzymatic digestion. Uterus fragments, 2–3 mm in size, were created using a scalpel and digested with 0.25% Trypsin–EDTA for 35 min at 37°C, followed by dissociation buffer (1 mg ml^–1^ collagenase, 1 mg ml^–1^ Dispase, 400 μg ml^–1^ DNase I) for 45 min at 37°C. Cell clumps were homogenized by passage through a 22-gauge syringe followed by passage through a 40-μm nylon mesh filter to remove remaining clumps. For all species except opossum, a single-cell suspension was obtained from the lysate, transferred to fresh growth medium and cultured in T25 flasks. To facilitate enrichment of fibroblasts versus epithelial cells, media were exchanged in each well after 15 min to remove floating cells that had not yet attached while stromal fibroblasts had attached. Cells were grown to confluency and sub-passaged by scraping the cells off the surface to be split into two T25 flasks. For opossum, the single-cell suspension was layered onto a Percoll^®^ density gradient for further separation. Immunohistochemistry was used to test for abundance of vimentin (Santa Cruz, sc-6260) and cytokeratin (Abcam, ab9377) to validate fibroblast subtype in the isolated cells.

### Cell culture

ESFs were grown in phenol-red free DMEM/F12 with high glucose (25 mM), supplemented with 10% charcoal-stripped calf serum (Gibco) and 1% antibiotic/antimycotic (Gibco). BJ5ta (ATCC) cells were cultured in 80% DMEM and 20% MEM supplemented with 10% FBS, 1% antibiotic/antimycotic and 0.01 mg ml^–1^ hygromycin. SFs were cultured in DMEM with high glucose supplemented with 10% FBS.

### RNA isolation and sequencing

RNA was isolated using RNeasy micro kit (QIAGEN) and resuspended in 15 μl of water. The Yale Center for Genome Analysis ran samples on the Agilent Bioanalyzer 2100 to determine RNA quality, prepared mRNA libraries and sequenced on Illumina HiSeq2500 to generate 30–40 million reads per sample (single-end 75 base pair reads).

### Transcript-based abundances using RNAseq data

RNAseq data obtained were quantified using the transcript-based quantification approach as given in the program ‘kallisto’ [38]. Here, reads are aligned to a reference transcriptome using a fast hashing of k-mers together with a directed de Bruijn graph of the transcriptome. This rapid quantification technique produces transcript-wise abundances which are then normalized and mapped to individual genes and ultimately reported in terms of TPM [14, 15]. The Ensembl release 99 [39] gene annotation model was used and raw sequence reads (single-end 75 bp) for ESFs and SFs from human (*Homo sapiens*), cow (*Bos taurus*), dog (*Canis lupus*), cat (*Felis catus*), guinea pig (*Cavia porcellus*), horse (*Equus caballus*), monkey (*Macaca mulatta*), opossum (*Monodelphis domestica*), rabbit (*Oryctolagus cuniculus*), sheep (*Ovis aries*), rat (*Mus musculus*) and pig (*Sus scrofa*) were aligned to GRCh38.p13, ARS-UCD1.2, CanFam3.1, Felis_catus_9.0, Cavpor3.0, EquCab3.0, Mmul_10, MonDom5 (Release 97), OryCun2.0, Oar_v3.1, Rnor_6.0 and Sscrofa11.1 reference transcriptome assemblies. In order to facilitate gene expression across species, a one-to-one ortholog dataset consisting of 8639 species was formulated (comprising of human, rat, rabbit, horse, guinea pig, cat, sheep, cow, horse, dog and opossum) such that the sum of TPMs across these 8639 genes for each species totals to 10^6^. Additionally, the extended SF dataset comprising of pig and monkey was formulated and contained a total of 7743 one-to-one orthologs.

### Ancestral state reconstruction

Ancestral character estimation was performed on TPM values using the REML method available in the *ace* module of APE in R statistical package [40, 41].

### Isoform abundances using RNAseq data

The reads obtained as a result of RNA sequencing were quantified by adopting a transcript-based quantification approach. The program called Kallisto [38] was utilized to this end wherein the reads were aligned to an indexed reference transcriptome using a fast hashing of k-mers together with a directed de Bruijn graph of the transcriptome [42]. A list of transcripts that are compatible with a particular read are generated. This rapid quantification technique produces transcript-wise abundances and these were reported in terms of TPM [14, 15]. In order to identify splice variants from RNAseq data, we utilized transcript-based abundances obtained from Kallisto. A comprehensive literature survey on exon architecture of *CD44* was followed by mapping the Kallisto-obtained abundances to reported *CD44* isoforms in order to verify which splice variants are expressed.

### Protein abundances

We used a proteomic method called data-independent acquisition mass spectrometry (DIA-MS) [43] to quantify the ratio of CD44 proteins from each sample to the total proteomes. Compared to traditional shotgun proteomics, DIA-MS considers and matches both MS1 (peptide)- and MS2 (peptide fragment)-level ion traces, both of high m/z resolution, along with the liquid chromatography (LC) separation for peptide/protein identification [44]. The implementation of DIA-MS was identical to published [45]. Briefly, the human and cow SF cell samples were washed, harvested and snap-frozen by liquid nitrogen. The protein extraction was performed by adding 10 M urea containing complete protease inhibitor cocktail (Roche) and Halt™ Phosphatase Inhibitor (Thermo) and digested. About 1.5 μg of peptides from each sample were used for DIA-MS measurement on an Orbitrap Fusion Lumos Tribrid mass spectrometer (Thermo Scientific) platform coupled to a nanoelectrospray ion source, as described previously [45]. DIA-MS data analyses were performed using Spectronaut v13 [46], by searching against the UniProt proteome databases of *H. sapiens* (human) and *B. taurus* (cow) separately for samples of different species. Both peptide and protein FDR cutoff (Qvalue) were controlled at 1%, and the label-free protein quantification was performed using the default settings in Spectronaut. Experimental replicates cattle SF = 3, human SF = 5. A few peptides of CD44 were manually checked with their DIA LC-peak abundances in Spectronaut for the relative quantification between human and cow species.

#### 5′-RACE

RNA was isolated by RNeasy mini kit (Qiagen) from BJ5ta cells, bovine dermal fibroblasts, human ESFs and bovine endometrial cells. RNA was purified via phenol–chloroform extraction. The mRNA was enriched using the MagJET mRNA Enrichment Kit (Thermo Scientific). Then, the 5′-RACE procedure was performed using the FirstChoice^®^ RLM-RACE Kit (Invitrogen™). cDNA containing the 5′ UTR of CD44 from each cell type was amplified using a nested PCR strategy with the primers:

Arbitrary outer, forward adapter (both human and bovine CD44): GCTGATGGCGATGAATGAACACTG

Arbitrary inner, forward adapter (both human and bovine CD44): ACTGCGTTTGCTGGCTTTGATG

Human CD44 outer, reverse: GGAGGTGTTGGATGTGAGGATGTA

Human CD44 inner, reverse: CATTGTGGGCAAGGTGCTATTG

Bovine CD44 outer, reverse: GGAGGTGTTGGATGTGAGGATGTA

Bovine CD44 inner, reverse: ATGGTGGGCAGCGTGCTATTA

PCR products were run on 2% SDS-polyacrylamide gels, gel-extracted, sequenced and aligned to the human or bovine genome to elucidate the 5′-UTR length and, ultimately, the transcription start site of CD44 in each cell type.

### Analysis of CRE

We searched for putative CRE that may affect *CD44* gene expression in the different species. Position-specific frequency matrix motifs of known eukaryotic transcription factor binding sites were downloaded from the JASPAR database [47]. The genome sequences of the species were obtained from the Ensembl database: https://doi.org/10.1093/nar/gkz966. We considered genomic regions 5 kb upstream to and 1 kb downstream of the translation start site of each gene as the promoter regions to be analyzed. Alignment to the binding site motifs within the CD44 promoter region in each species was calculated using the FIMO package using default parameters [48]. We considered valid matches where the alignment was reported to be statistically significant with a *P*-value < 10^−4^. CEBPB binding sites were then selected for further analysis.

### Plasmids and constructs

The promoter fragment (−2679 ± 406) of the human *CD44* was PCR amplified from chromosomal DNA of Human ESF cells with primers introducing XhoI (5′-GCCGCTCGAGAGGTTCCATGAAACACAGTAAGA-3′) or HindIII(5′-CCCAAGCTTGCGAAAGGAGCTGGAGGAT-3′) restriction sites. The cow CD44 promoter fragment (−2886 ± 42) was PCR amplified from chromosomal DNA of Cow ESF cells with primers introducing XhoI (5′-CCGCTCGAGCTGCTAAGTCGCTTCAGTCAT-3′) or HindIII(5′-AGCCCAAGCTTGGAAGTTGGGTGCAGTTTTT-3′) restriction sites. The resulting fragment was cloned into a Promoterless NanoLuc^®^ Genetic Reporter Vectors pNL2.1 [Nluc/Hygro] (Promega), and sequence was verified.

### Transfection assays

For *CD44* promoter analysis, cells in 24-well plates were co-transfected with 200 ng human or cow *CD44*-promoter-pNL2.1 or empty vector pNL2.1, and 40 ng pGL4.13 with Lipofectamine 3000 (Invitrogen) according to the manufacturer. Cells were lysed 24 h after transfection for luciferase assay with the Nano-Glo Dual-Luciferase^®^ Reporter Assay System (Promega). *CD44* promoter activity (NanoLucR luciferase) was normalized with firefly luciferase activity.

The transfection experiments with ESF were replicated eight times, those with SF were replicated five times. The higher number of replicates with ESF was in response to the higher variance of the results compared to SF.

### Real-time PCR

Cells in six-well plates were transfected with 1 μg human or cow *CD44*-promoter-pNL2.1 or empty vector pNL2.1 using Lipofectamine 3000 (Invitrogen) as manufacturer’s instructions. Total RNAs from cells were isolated using RNeasy Plus Micro Kit (QIAGEN) according to the manufacturer’s protocol. After digestion by RNase-Free DNase Set (QIAGEN), RNAs were reverse-transcribed according to iScript cDNA Synthesis Kit (Thermo Fisher Scientific). Real-time polymerase chain reaction (PCR) was performed using an Applied Biosystems™ Fast SYBR™ Green Master Mix (Thermo Fisher Scientific). Primer sequences used for real-time PCR are Nluc-1F: CAGGGAGGTGTGTCCAGTTT, Nluc-1R: TCGATCTTCAGCCCATTTTC for evaluation of *CD44*-promoter-driven Luciferase activity; Hygr-1F: GAGCCTTCAGCTTCGATGTC, Hygr-1R: CGGTACACGTAGCGGTCTTT for evaluation of the expression of hygromycin driven by constitutive promoter. TBP expression was used for normalization. The 2^−ΔΔCt^ method was used for data analysis.

For detecting endogenous cow and human *CD44* expression, the primer sequences used for real-time PCR were listed as follows:

Bt-CD44-Forwad: TACAGCATCTTCCACACGCA

Bt-CD44-Reverse: GCCGTAGTCTCTGGTATCCG

Bt-TBP-2F: GCACAGGAGCCAAGAGTGAA,

Bt-TBP-2R: TTCACATCACAGCTCCCCAC

Hs-CD44-1F: GATGGAGAAAGCTCTGAGCATC

Hs-CD44-1R: TTGCTGCACAGATGGAGTTG

Hs-TBP-1F: GGAGAGTTCTGGGATTGTAC

Hs-TBP-1R: CTTATCCTCATGATTACCGCAG

TBP was used for normalization.

### Cell culture, RNA-interference and quantitative PCR

Human Skin Fibroblasts (BJ-5ta, ATCC CRL-4001) were cultured in a 4:1 mixture of four parts Dulbecco’s Modified Eagle’s Medium containing 4 mM L-glutamine, 4.5 g/L glucose and 1.5 g/L sodium bicarbonate and one part Medium 199 supplemented with 0.01 mg/ml hygromycin B, and 10% fetal bovine serum.

Human Endometrial Stromal Fibroblasts (T-HESCs, ATCC CRL-4003) were cultured in a 1:1 mixture of Dulbecco’s Modified Eagle’s medium and Ham’s F-12 medium with 3.1 g/L glucose and 1 mM sodium pyruvate without phenol red (D2906, Sigma) supplemented with 1.5 g/L sodium bicarbonate, 1% ITS+ Premix (354352, BD), 1% ABAM and 10% Charcoal stripped fetal bovine serum (100–119, Gemini).

Twelve-well plates were grown to 70% confluency and transfected with 25 nmol final concentration of siRNAs targeting *CEBPB* (s2891 and s2892 Themo Fisher). In preparation for transfection, siRNAs in OptiMem I Reduced Serum Media (31985, Thermo Fisher) were mixed with an equal volume of OptiMem containing Lipofectamine RNAiMax (13778, Thermo Fisher), incubated at room temperature for 20 min, and added dropwise to cells in 1 ml growth media. Final concentration of siRNAs was 25 nM. Control wells were prepared without any siRNA added.

The data on the KD effects of CEBPB are based on four experimental replicates.

Knockdown of *CEBPB* and expression of *CD44* were confirmed by qPCR. Media was removed, cells were washed in PBS followed by direct lysis with Buffer RLT Plus + beta-mercaptoethanol. RNA was extracted according to the manufacturer’s protocol (74034, RNeasy Plus Micro Kit, Qiagen). Reverse transcription of 1 µg of RNA was carried out with iScript cDNA Synthesis Kit (1708891, Bio-Rad) using an extended transcription step of 3 h at 42°C. qPCR reactions were with Taqman Fast Universal PCR Master Mix (4366072, Applied Biosystems) in duplicate using 5 ng of cDNA for template each. Taqman probes for *CEBPB* (hs00270923_s1, Invitrogen) and *CD44* (Hs01075864_m1, Invitrogen) were used to amplify each template. Fold change was calculated by finding the ddCt values relative to the expression of TATA Binding Protein.

## SUPPLEMENTARY DATA

Supplementary data is available at *EMPH* online.

## FUNDING

This study was funded by the National Cancer Institute U54 grant CA209992 for the Cancer Systems Biology Center at Yale.

**Conflict of interest:** None declared.

## Supplementary Material

eoac036_Supplementary_Data
