# Supplementary material for: Evolution of higher mesenchymal CD44 expression in the human lineage: A gene linked to cancer malignancy
Source: Evol Med Public Health. 2022 Aug 30;10(1):447–62. doi: 10.1093/emph/eoac036 (PMC9487634; doi:10.1093/emph/eoac036)
Supplement: eoac036_Supplementary_Data [file eoac036_supplementary_data.zip › CD44 Suppl Figures and table V8 with captions.pdf]

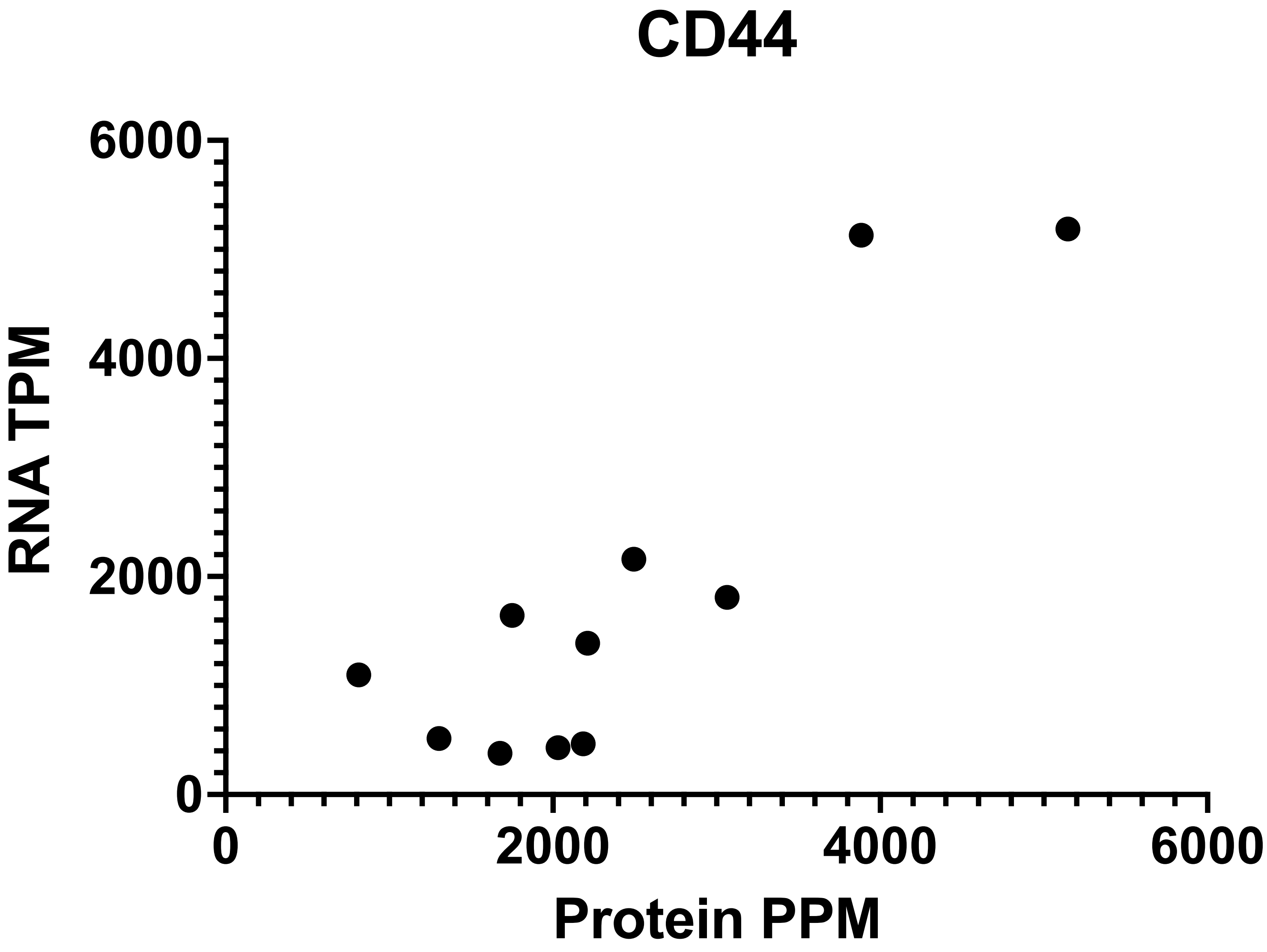

Suppl. Figure 1: correlation between CD44 protein abundance and RNA expression in skin fibroblasts cross 11 mammalian species. The Spearman correlation is 0.7545.

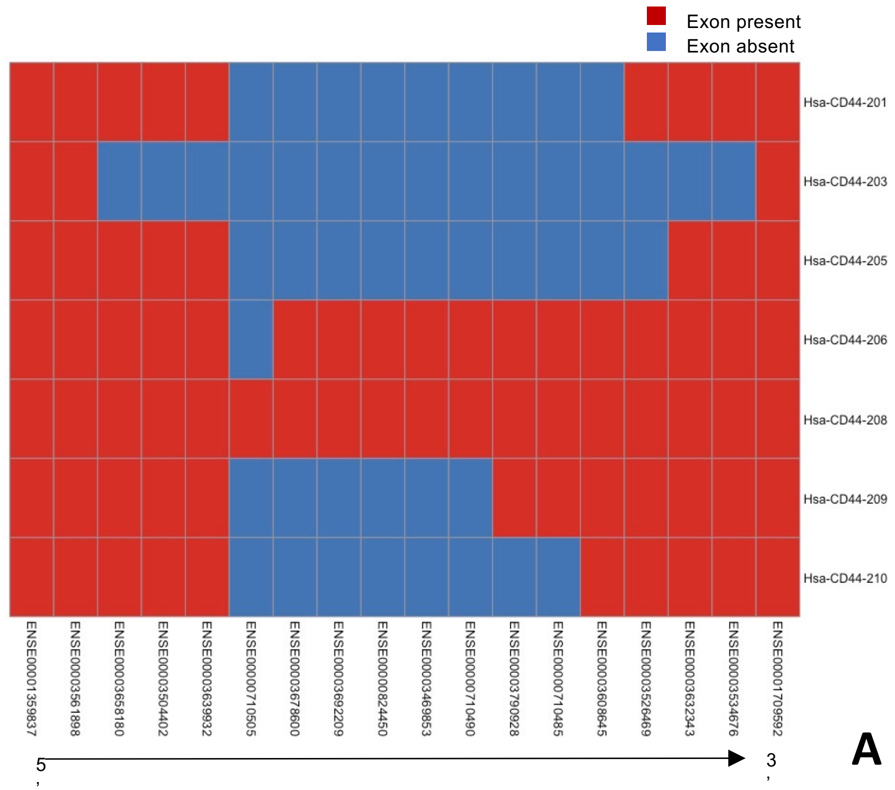

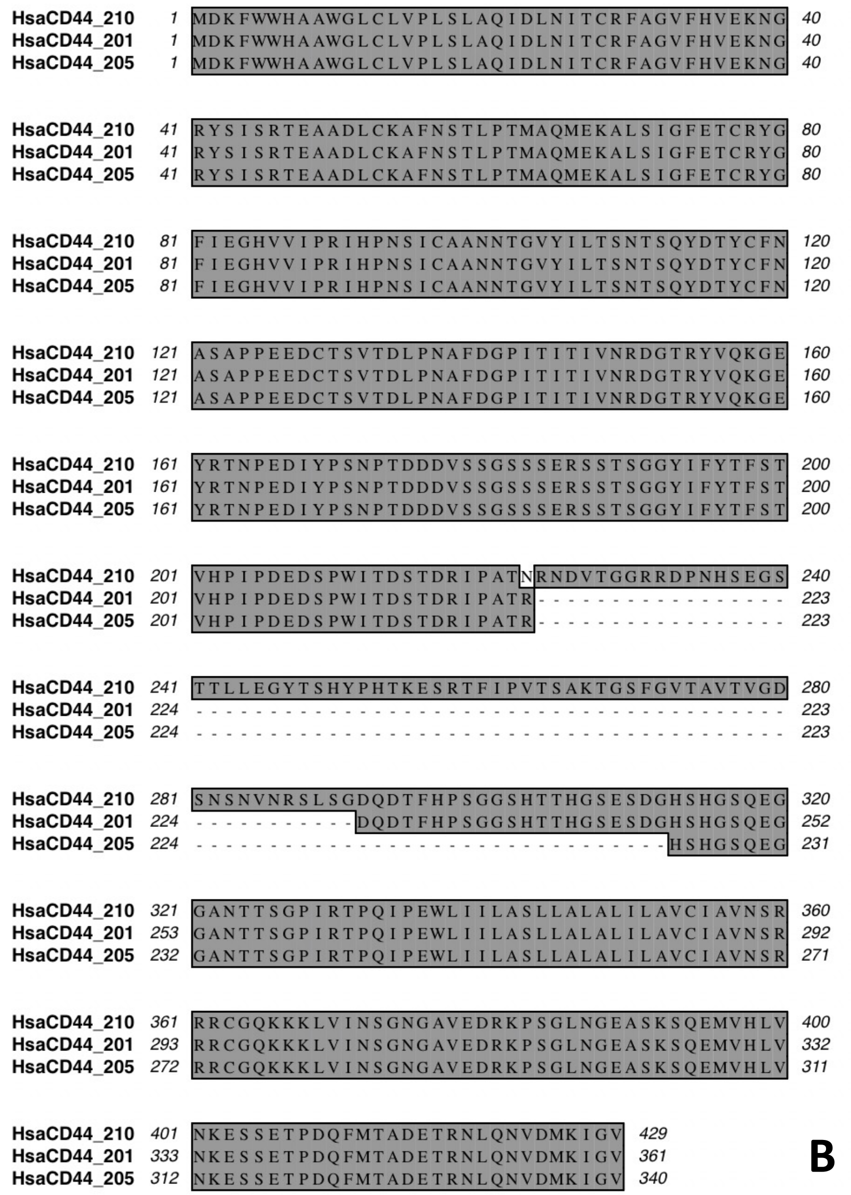

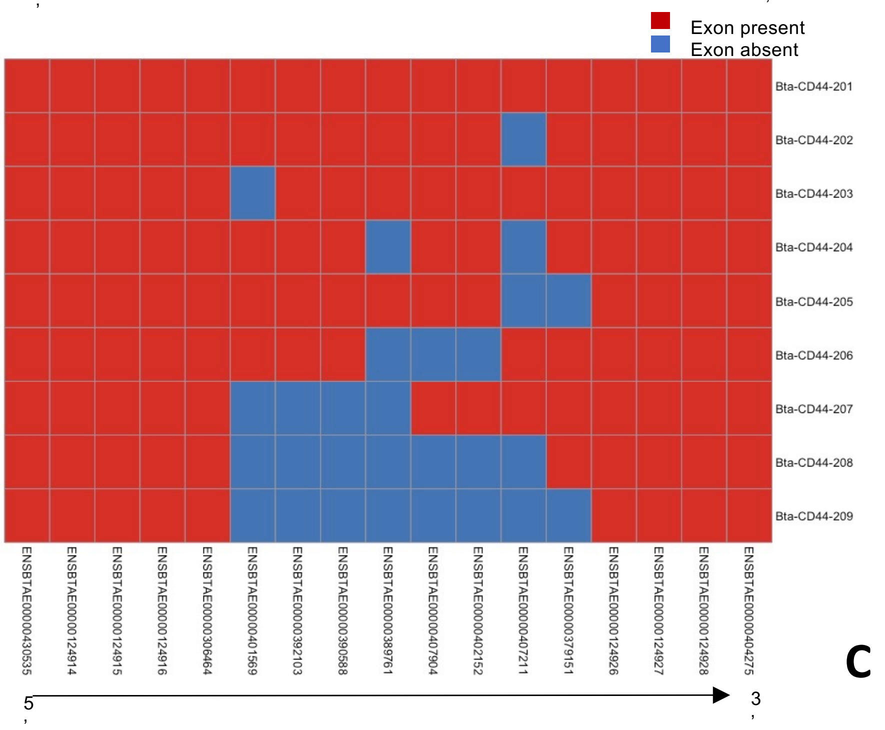

Suppl. Figure 2: Exon composition of CD44 transcripts. A) Exon composition of transcripts obtained from human SF and ESF RNA. B) Alignment of the computational translation of transcripts of the three dominant isoforms. The alignment shows that the different identities of the 3' and 5' most exons does not affect the amino acid sequence. C) Exon composition of transcripts identified from RNAseq data from cow mesenchymal cells.

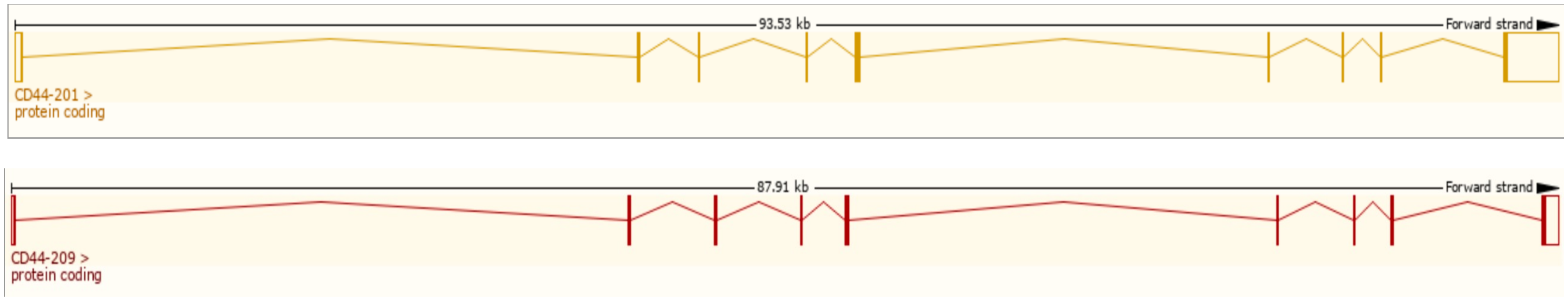


```
HsaCD44_201    1  MDKFWWHAAWGLCLVPLSLAQIDLNITCRFAGVFHVEKNG   40
BtaCD44_209    1  MDTFWWRAAWGLCLVQLSLAQIDLNITCRYAGVFHVEKNG   40

HsaCD44_201   41  RYSISRTEAADLCKAFNSTLPTMAQMEKALSIGFETCRYG   80
BtaCD44_209   41  RYSISKTEAADLCKAFNSTLPTMAQMEAARNIGFETCRYG   80

HsaCD44_201   81  FIEGHVVIPRIHPNSICAANNTGVYILTSNTSQYDTYCFN  120
BtaCD44_209   81  FIEGHVVIPRIHPNSICAANNTGVYILTSNTSQYDTYCFN  120

HsaCD44_201  121  ASAPPEEDCTSVTDLPNAFDGPITITIVNRDGTRYVQKGE  160
BtaCD44_209  121  ASAPPGEDCTSVTDLPNAFEGPITITIVNRDGTRYTKKGE  160

HsaCD44_201  161  YRTNPEDIY-----PSNPTDDDVSSGSSSERSSTSGGYIF  195
BtaCD44_209  161  YRTNPEDINPSVVSPSSPPDDEMSSGSPSERSTSGGYSIF  200

HsaCD44_201  196  YT-FSTVHPIPDEDSPWITDSTDRIPATRDQDTFH-PSGG  233
BtaCD44_209  201  HTHLPTVHPSPDQDGPWVSSEPENTSDTRDYGSSHDPSGR  240

HsaCD44_201  234  SHTTHGSESDGHSHGSQEGGANTTSGPIRTPQIPEWLIIL  273
BtaCD44_209  241  SYTTHASESAGHSSGSEEHGANTTSGPMRKPQIPEWLIIL  280

HsaCD44_201  274  ASLLALALILAVCIAVNSRRRCGQKKKLVINSGNGAVEDR  313
BtaCD44_209  281  ASLLALALILAVCIAVNSRRRCGQKKKLVINNGNGTMEER  320

HsaCD44_201  314  KPSGLNGEASKSQEMVHLVNKESSETPDQFMTADETRNLQ  353
BtaCD44_209  321  KPSGLNGEASKSQEMVHLVNKGSSETQDQFMTADETRNLQ  360

HsaCD44_201  354  NVDMKIGV  361
BtaCD44_209  361  NVDMKIGV  368
```

Suppl. Figure 3: homology of HsaCD44-201 and BtaCD44-209. These transcripts consist or 9 exons and correspond to the human CD44s and the protein ID P16070-13 aka CD44R4.

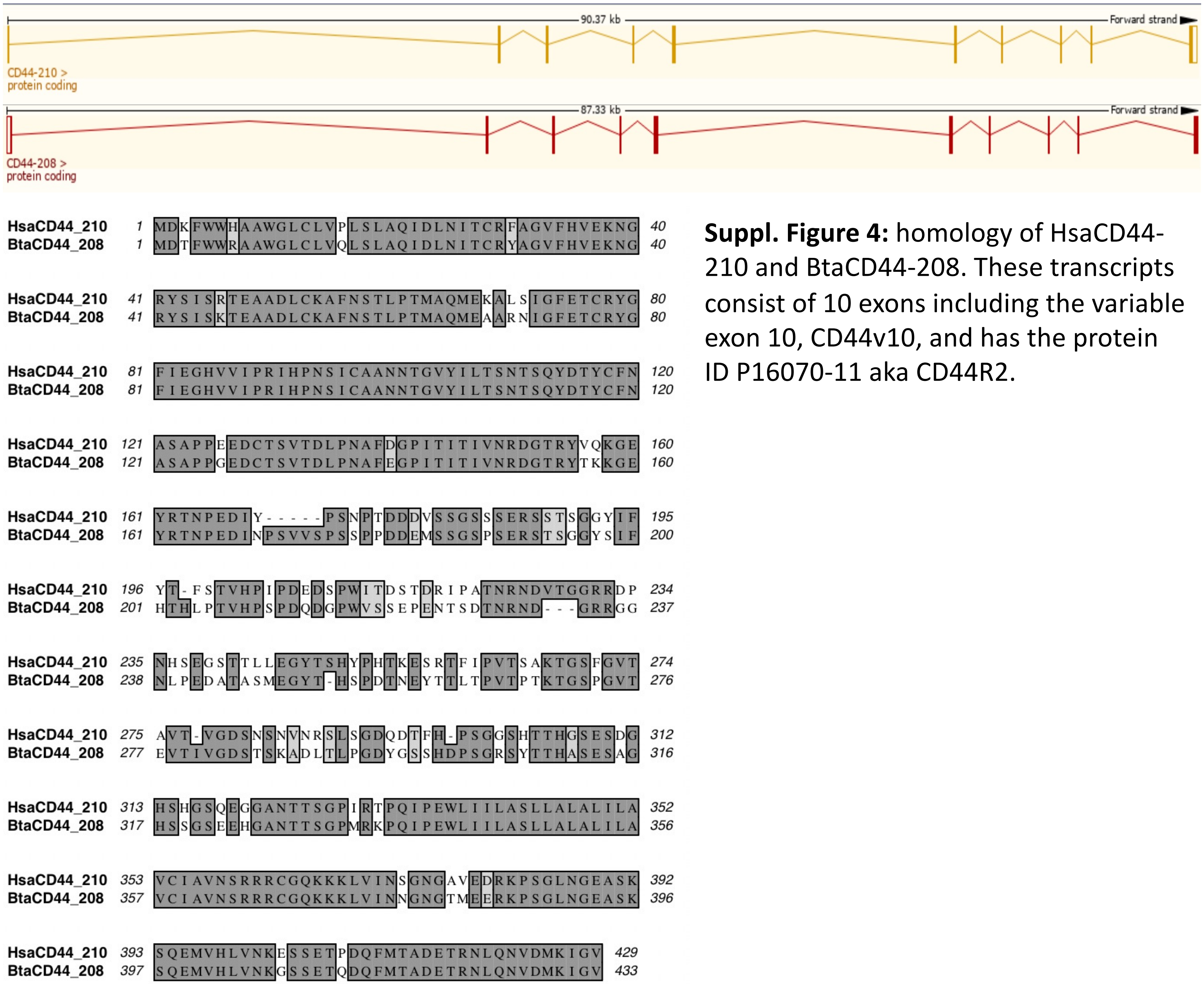

Suppl. Figure 4: homology of HsaCD44-210 and BtaCD44-208. These transcripts consist of 10 exons including the variable exon 10, CD44v10, and has the protein ID P16070-11 aka CD44R2.

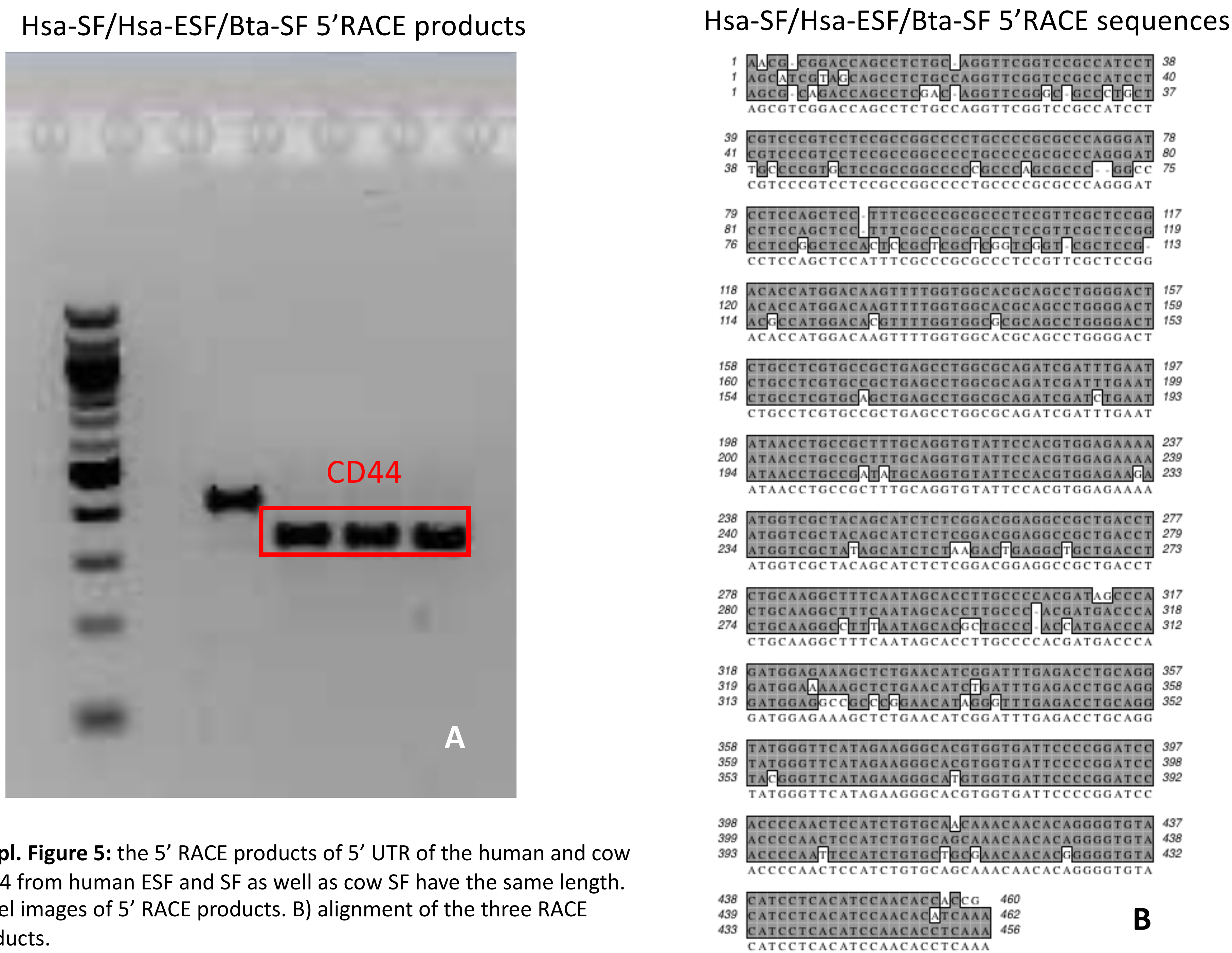

Suppl. Figure 5: the 5' RACE products of 5' UTR of the human and cow CD44 from human ESF and SF as well as cow SF have the same length. A) gel images of 5' RACE products. B) alignment of the three RACE products.

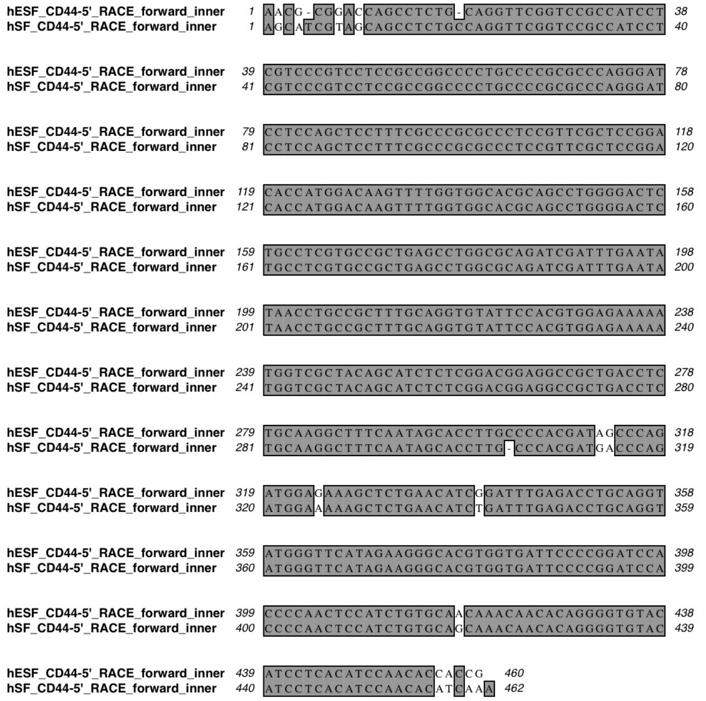

A

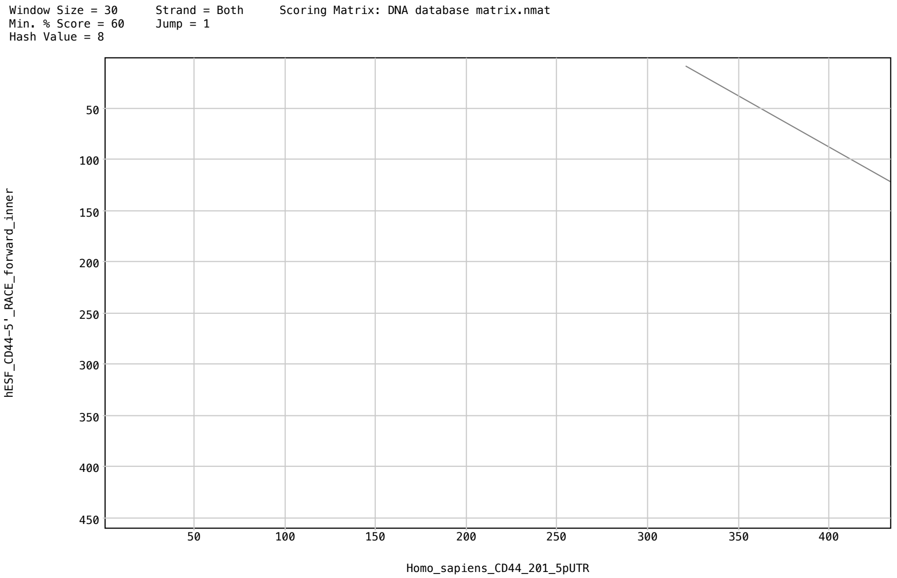

B

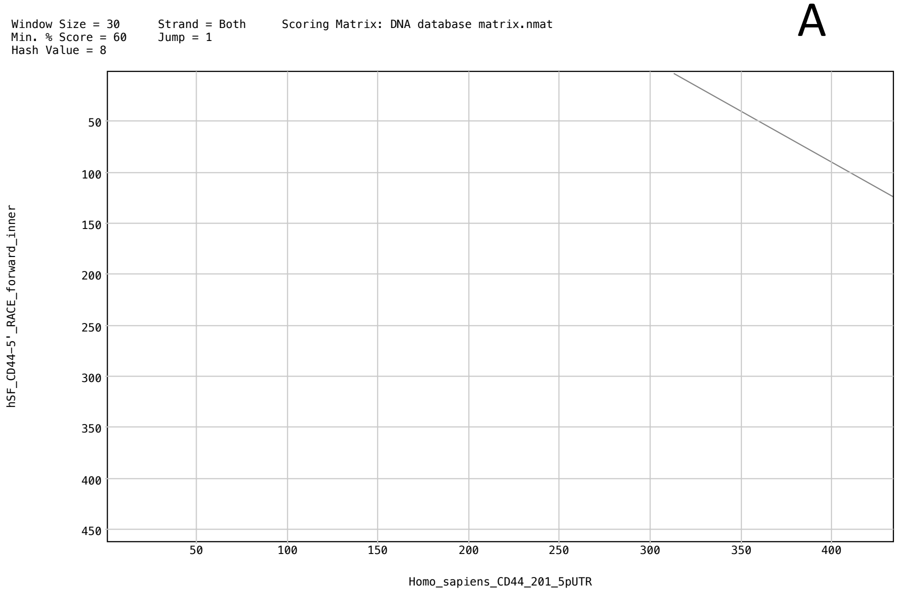

C

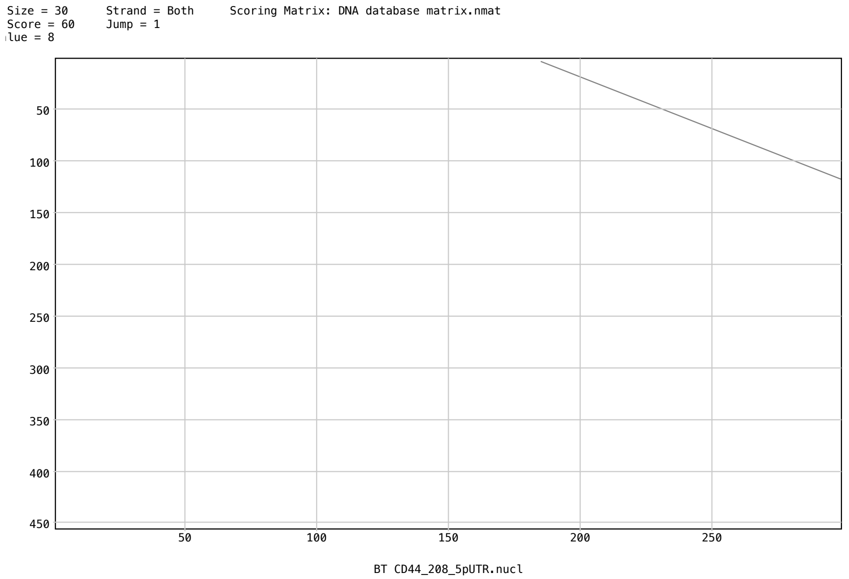

D

Suppl. Figure 6: comparison of the human 5’ RACE products with the 5’ exons in ENSEMLE. A) alignment of 5’ RACE products from human SF and ESF. B) HsaCD44-201 5’ UTR vs. the RACE product from human ESF. C) HsaCD44-201 5’ UTR vs. the RACE product from human SF. D) dotplot of BtaCD44-208 5’ UTR vs. the RACE product from bovine SF.

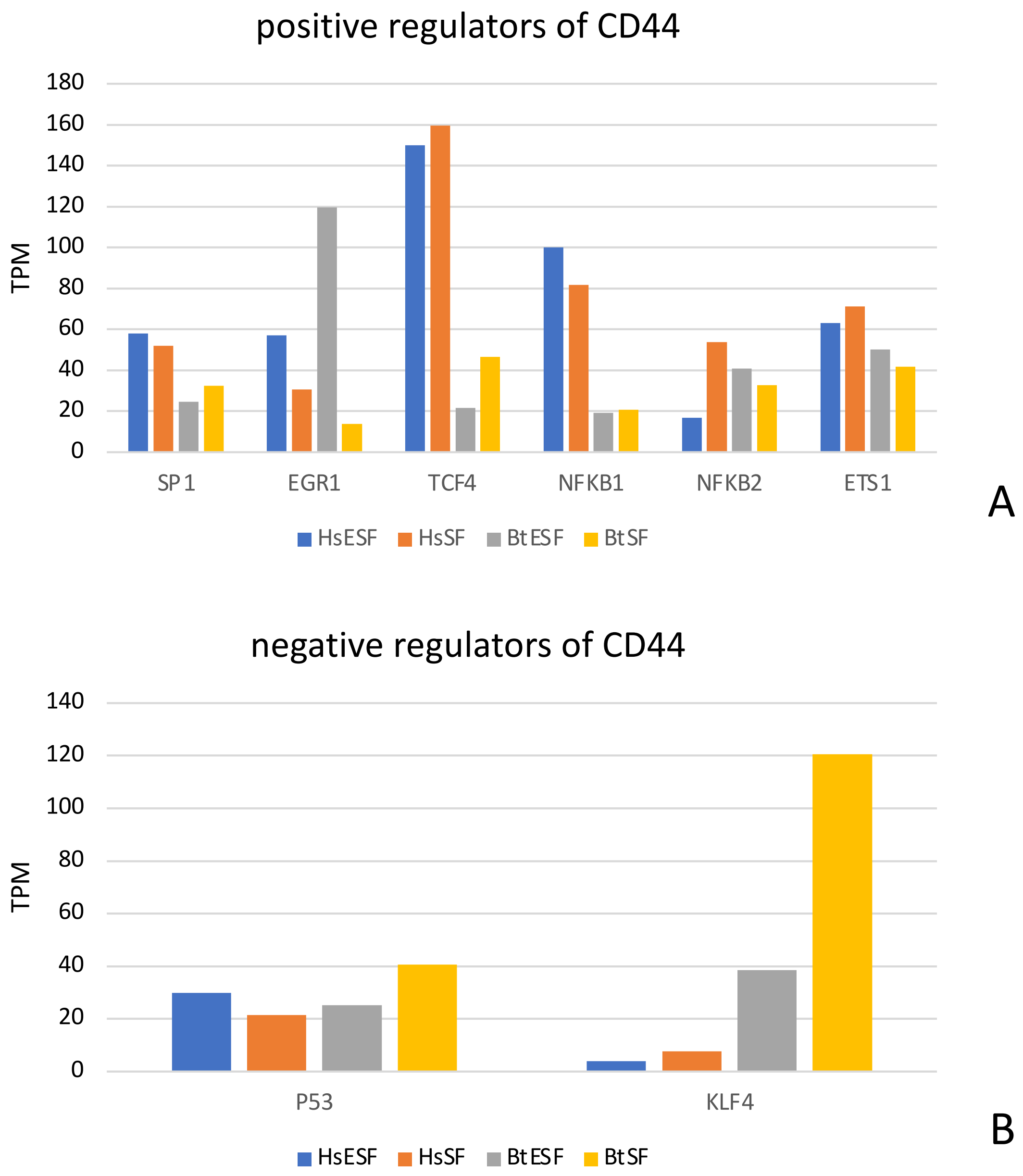

Suppl. Figure 7: transcription factors known to be regulators of *CD44* in cancer cells. A) RNA expression levels of transcription factors known to be positive regulators of *CD44* in cancer cells. B) RNA expression levels of transcription factors known to be negative regulators of *CD44* in cancer cells.

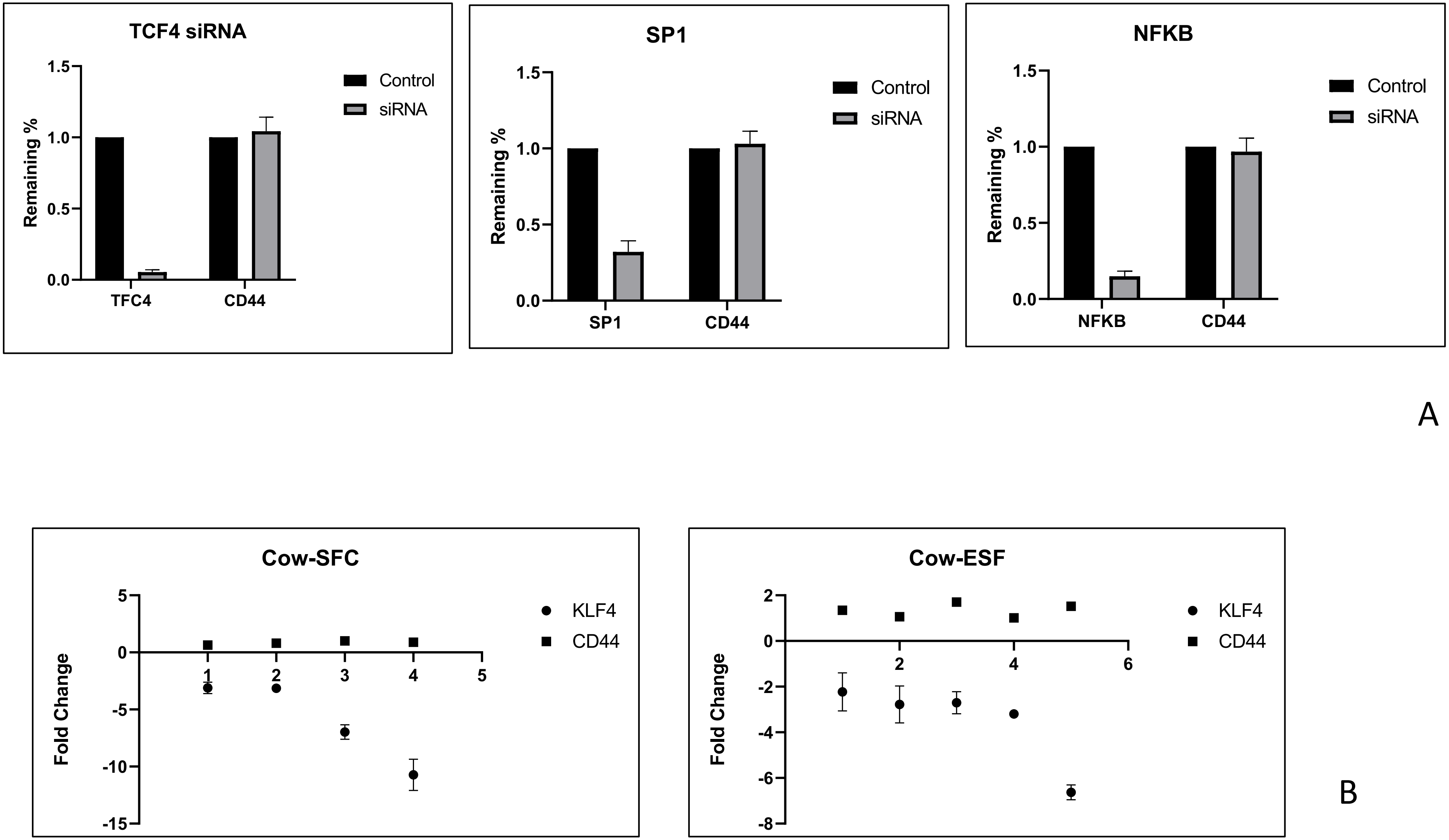

Suppl. Figure 8: siRNA mediated knockdown of regulators of CD44 which could explain increased expression in humans compared to cow. A) KD results with putative positive regulators of *CD44* in human cells. No effects have been detected. B) KD results with putative negative regulators of *CD44* in cow cells with different amounts of siRNA. No effects have been detected.

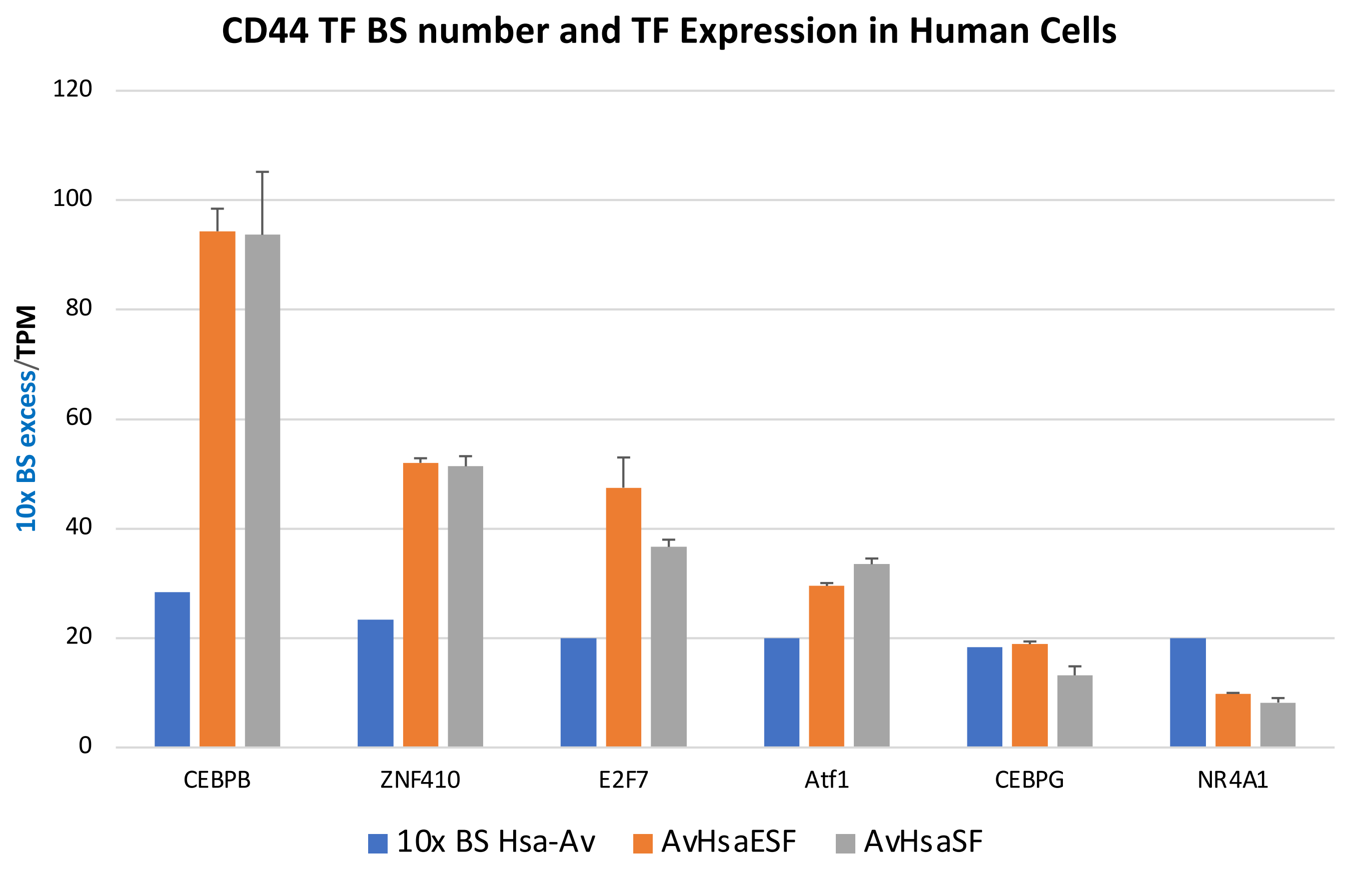

Suppl. Figure 9: abundance of binding sites and expression levels of cognate transcription factors in human ESF and SF. Blue bars are 10x number of binding sites at the human CD44 promoter, red bar expression level of TF [TPM] in human ESF and gray bar expression level of TF [TPM] in human SF.

Supplemental Table 1: experimentally verified transcription factors regulating *CD44* expression in humans with the location of their binding sites relative to the TSS of *CD44*. The references are from Chen et al., 2018, *J. Hematol. & Oncology* **11**:64.

Transcription factor	Location of binding site (bp upstream of TSS)	Reference (as in Chen et al. 2018)
**ETS1**	<-1400	Zhang et al. 2013
**P53**	-237 to -263	Godar et al., 2008
**EGR1**	<-301	Maltzuran et al., 1996
**FOXP3**	<-810	Zhang et al., 2015
**KLF4**	<-1420	Yan et al., 2016
**SALL4**	<-773	Yuan et al., 2016

Supplemental Table 2: Transcription factor binding site numbers at the CD44 promoter region as well as RNA expression levels of the corresponding transcription factor in human mesenchymal cells in TPM. Note that CEBPB has the most consistent difference in terms of binding site numbers of human relative to other species and the highest expression level in human cells and is thus the strongest candidate to explain part of the lineage specific expression levels of *CD44*.

	Transcription Factor Binding Site Numbers							TF [TPM]	
**TF**	Human	Rabbit	Guinea Pig	Rat	Horse	Sheep	Cow	AvHsaESF	AvHsaSF
**CEBPB**	3	0	1	0	0	0	0	94.32	93.67
**ZNF410**	3	1	1	0	1	0	1	52.01	51.37
**E2F7**	3	1	2	1	2	0	0	47.47	36.65
**Atf1**	2	0	0	0	0	0	0	29.51	33.48
**CEBPG**	2	0	1	0	0	0	0	18.87	13.18
**NR4A1**	3	1	1	1	2	1	0	9.82	8.20
